# Near-infrared light-responsive upconversion substrate enables spatiotemporal control of mesenchymal stem cells adhesion and multilineage differentiation in vivo

**DOI:** 10.1016/j.mtbio.2025.102696

**Published:** 2025-12-26

**Authors:** Jinming Li, Qingxin Zhao, Jiani Sun, Hao Zeng, Anli Yang

**Affiliations:** a MOE Key Laboratory of Laser Life Science & Institute of Laser Life Science, Guangdong Provincial Key Laboratory of Laser Life Science, Guangzhou Key Laboratory of Spectral Analysis and functionalized Probes, College of Biophotonics, School of Optoelectronic Science and Engineering, South China Normal University, Guangzhou, 510631, China; b Department of Breast Oncology, Sun Yat-sen University Cancer Center, State Key Laboratory of Oncology in South China, Guangdong Provincial Clinical Research Center for Cancer, Guangzhou, 510060, China

**Keywords:** Near-infrared light, Upconversion substrate, Spatiotemporal control, Mesenchymal stem cells, Cell adhesion and multilineage differentiation

## Abstract

Spatiotemporally precise control of mesenchymal stem cells (MSCs) differentiation remains an unmet challenge in regenerative medicine. Herein, we report a near-infrared (NIR)-responsive platform that integrates upconversion nanoparticle (UCNP)-functionalized substrates with host-guest photoresponsive chemistry (β-cyclodextrin/arylazopyrazole-RGD) to NIR spatiotemporally orchestrate MSCs adhesion, spreading, and lineage commitment with spatiotemporal precision. The UCNPs convert 808 nm NIR light into localized UV emissions, dynamically modulating azobenzene-cyclodextrin interactions and thereby tuning surface RGD ligand density via photoisomerization. Under low NIR intensities (0–0.5 W/cm^2^), augmented integrin-RGD binding drives robust focal adhesion assembly, actin cytoskeletal tension, nuclear YAP translocation, and osteogenic differentiation, as evidenced by 2–3 fold upregulation of BMP2, RUNX2, and ALP. Conversely, high-intensity NIR (1–2 W/cm^2^) induces RGD detachment, leading to suppressed cytoskeletal contractility and nuclear Lamin A/C expression, consequent cytoplasmic YAP retention, and activation of PPARγ-mediated adipogenesis, marked by 4–5 fold induction of PPARγ, C/EBPα, and FABP4. Most importantly, in vivo murine studies validate the platform's capacity for non-invasive, light-guided spatiotemporal regulation of MSCs adhesion, spreading, YAP signaling, and lineage specification (osteogenic vs. adipogenic), wherein differentiation thresholds (e.g., 1 W/cm^2^) correspond to clinically safe NIR exposure limits. This opto-material hybrid system effectively decouples photochemical stimulation to program MSC fate via mechanotransduction pathways, thereby providing a translatable strategy for the design of intelligent biomaterials towards personalized tissue regeneration.

## Graphical abstract

1. A near-infrared (NIR)-responsive upconversion platform controls surface RGD ligand density via host-guest chemistry. 2. NIR intensity spatiotemporally regulates the MSC mechanotransduction (YAP signaling) pathway in vivo*.* 3. Low NIR power induces osteogenic differentiation; high NIR power switches commitment to adipogenic differentiation.

**Figure:**
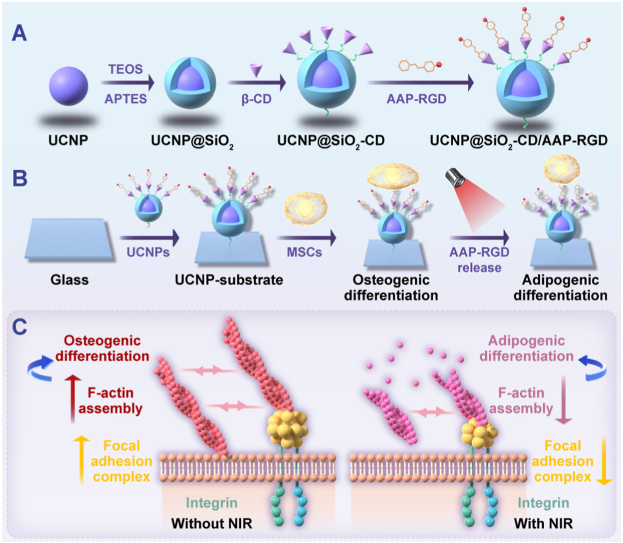

## 1 Introduction

Spatiotemporally precise control of mesenchymal stem cells (MSCs) differentiation remains a critical unmet need in regenerative medicine for treating skeletal disorders such as osteoporosis and osteoarthritis [1]. Conventional approaches, including soluble small molecules and growth factors, suffer from poor spatiotemporal resolution, off-target effects, potential toxicity, and carcinogenic risks [2,3]. Critically, recapitulating the dynamic and heterogeneous ligand presentation characteristic of the native extracellular matrix (ECM) within engineered biomaterials is essential for guiding complex cellular behaviors, including adhesion, mechanosensing, and ultimately, lineage commitment [4].

Cell adhesion, primarily mediated by integrin binding to adhesive ligands such as the Arg-Gly-Asp (RGD) peptide sequence, serves as a fundamental regulator of diverse cellular processes, including cytoskeletal organization, focal adhesion (FA) assembly, mechanotransduction signaling, and differentiation [5]. Consequently, developing biomaterials capable of NIR spatiotemporally and reversibly controlling ligand presentation offers a powerful strategy for mimicking ECM dynamics and regulating cell fate with spatiotemporal precision [6,7]. Light-responsive systems, employing molecules such as photolabile cages or photoisomerizable switches (e.g., azobenzene), have demonstrated temporal control over adhesion in vitro [8,9]. However, their reliance on ultraviolet (UV) or visible light results in severely limited tissue penetration and poses risks of phototoxicity, hindering in vivo translation. Alternative NIR spatiotemporal control strategies, such as magnetic field-mediated ligand movement (e.g., enabling macroscale spatial redistribution of ligands) or small-molecule-triggered reversible coordination switches (e.g., Mg^2+^-bisphosphonate), have achieved reversible manipulation of cell adhesion [10,11]. Nonetheless, these approaches often necessitate invasive procedures (e.g., implanting magnets, injecting chemical triggers) and have yet to fully recapitulate the complex, multilineage differentiation cascades observed in native tissues within a single, NIR spatiotemporally controllable platform in vivo.

Near-infrared (NIR) light (700–1000 nm), characterized by its deep tissue penetration and minimal phototoxicity, presents an attractive alternative for in vivo applications [[12], [13], [14]]. Lanthanide-doped upconversion nanoparticles (UCNPs) efficiently convert biocompatible NIR light into localized UV/visible emissions, thereby bridging the deep-tissue penetration advantage of NIR to the versatility of photochemical reactions at depth [[15], [16], [17]]. UCNP-functionalized substrates have facilitated NIR-controlled fundamental cell adhesion and detachment [[18], [19], [20]]. However, existing UCNP-based systems have fallen short of achieving the more sophisticated objective of NIR spatiotemporally guiding MSC multilineage differentiation, mimicking natural processes, within a living organism using NIR light alone.

**Scheme 1:**
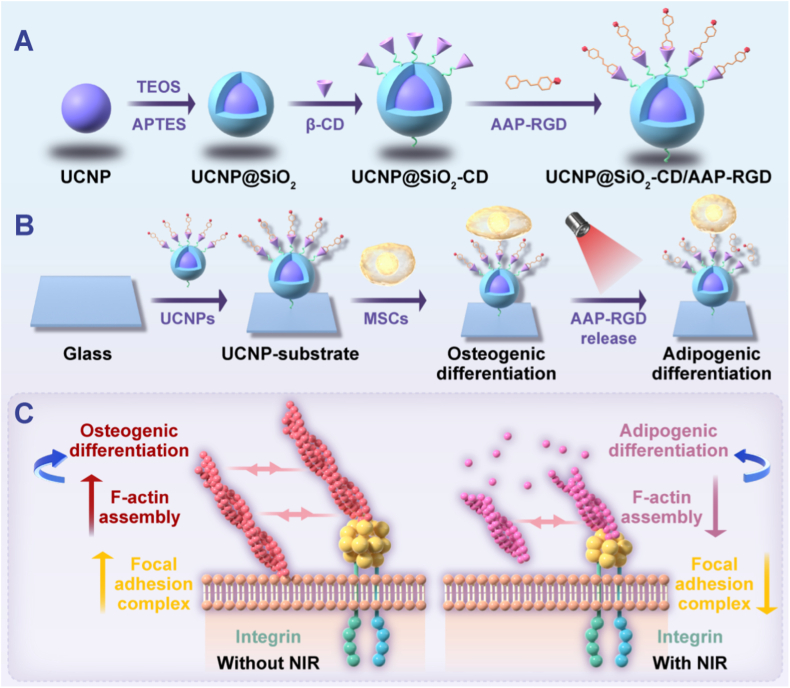
Schematic of the NIR-Responsive UCNP-Substrate Platform for Spatiotemporal Control of MSC Behavior. (A) Synthesis process of functionalized UCNPs (UCNP@SiO₂-CD/AAP-RGD) involving silica coating, β-cyclodextrin (β-CD) conjugation, and host-guest assembly with arylazopyrazole-modified RGD (AAP-RGD). (B) Assembly of the UCNP-substrate and mechanism of NIR light (808 nm)-triggered RGD release. UCNPs convert NIR to localized UV light, inducing AAP photoisomerization and dissociation from β-CD, thereby dynamically tuning surface RGD density. This controls MSC adhesion, cytoskeletal tension, YAP/TAZ signaling, and directs differentiation towards osteogenesis (low NIR power) or adipogenesis (high NIR power). (C) Proposed mechanotransduction pathway: High RGD density (Low NIR) promotes integrin ligation, robust focal adhesion (FA) assembly, actin stress fiber formation, nuclear stiffening (high Lamin A/C), nuclear YAP translocation, and activation of osteogenic programs (e.g., via RUNX2). Low RGD density (High NIR) suppresses FA formation, cytoskeletal tension, and nuclear mechanics, leading to cytoplasmic YAP retention and activation of adipogenic programs (e.g., via PPARγ).

Herein, we report a novel NIR-responsive platform that integrates UCNP-functionalized substrates with host-guest photoresponsive chemistry (specifically, β-cyclodextrin (β-CD) and arylazopyrazole-modified RGD (AAP-RGD)) to achieve non-invasive, spatiotemporal, and reversible control over MSC adhesion, spreading, and multilineage differentiation both in vitro and in vivo (Scheme 1). The UCNP substrate converts 808 nm NIR light into localized UV emissions, triggering the photoisomerization of AAP. This dynamically modulates the host-guest interaction between AAP and β-CD, thereby reversibly controlling the surface density of the bioactive RGD ligand. Crucially, this design allows decoupling photochemical cues for programming downstream cellular mechanotransduction and fate specification. We hypothesized that modulating NIR intensity would tune RGD density, thereby regulating integrin ligation, FA assembly, cytoskeletal tension, nuclear mechanics (e.g., YAP/TAZ localization), and ultimately directing MSC commitment toward osteogenic or adipogenic lineages.

In this study, we demonstrate: 1. Successful synthesis and characterization of the UCNP@SiO_2_-CD/AAP-RGD substrate and its NIR-responsive RGD release kinetics. 2. That in situ NIR irradiation dynamically regulates MSC adhesion, spreading, focal adhesion assembly, actin cytoskeleton organization, nuclear morphology/stiffness (lamin A/C), and YAP/TAZ subcellular localization in a power-dependent manner in vitro. 3. That distinct NIR intensities (low: 0–0.5 W/cm^2^; high: 1–2 W/cm^2^) steer MSC differentiation toward osteogenesis or adipogenesis, respectively, via modulating the mechanotransduction-YAP axis, and that this differentiation exhibits temporal controllability. 4. The platform's efficacy in enabling non-invasive, light-guided spatiotemporal regulation of MSC adhesion, spreading, YAP signaling, and osteogenic/adipogenic differentiation in vivo, while operating within clinically safe NIR power thresholds (e.g., 1 W/cm^2^). This opto-material hybrid system offers a translatable strategy for intelligent biomaterial design in personalized tissue regeneration.

While our group has previously reported a UCNP-based platform for controlling MSC differentiation [21], that system was based on an irreversible photocleavage mechanism, which functions as a terminal, binary switch. The current work represents a significant conceptual and technological advancement by employing a dynamic and, in principle, reversible host-guest chemistry based on arylazopyrazole photoisomerization. This advanced mechanism operates like an analog dial, enabling a more sophisticated and tunable method of controlling ligand availability that better mimics the dynamic nature of the native ECM. Furthermore, building upon our previous findings, we provide a much deeper mechanistic insight into the downstream mechanobiological cascade. We elucidate the crucial role of the nuclear lamina protein Lamin A/C as a mechanostat and provide a more complete analysis of the focal adhesion-cytoskeleton-YAP axis, thereby establishing a more detailed pathway from the initial photonic input to the ultimate cell fate decision.While the individual signaling components, such as integrin-mediated adhesion and the YAP/TAZ mechanotransduction cascade, are well-understood, the novelty of this work lies in the development of an advanced opto-material platform that provides an external, non-invasive handle to precisely control this complex cellular machinery. Our objective was not to uncover new biological pathways, but rather to engineer a sophisticated tool that can hijack these known pathways for therapeutic ends. By demonstrating that the intensity of biocompatible NIR light can be used to quantitatively steer MSC lineage commitment in vivo, we provide a proof-of-concept for a new class of ‘smart’ biomaterials capable of directing tissue regeneration with unprecedented spatiotemporal precision.

## 2 Results and discussion

### 2.1 Synthesis, characterization and performance test of UCNP-substrate

**Fig. 1:**
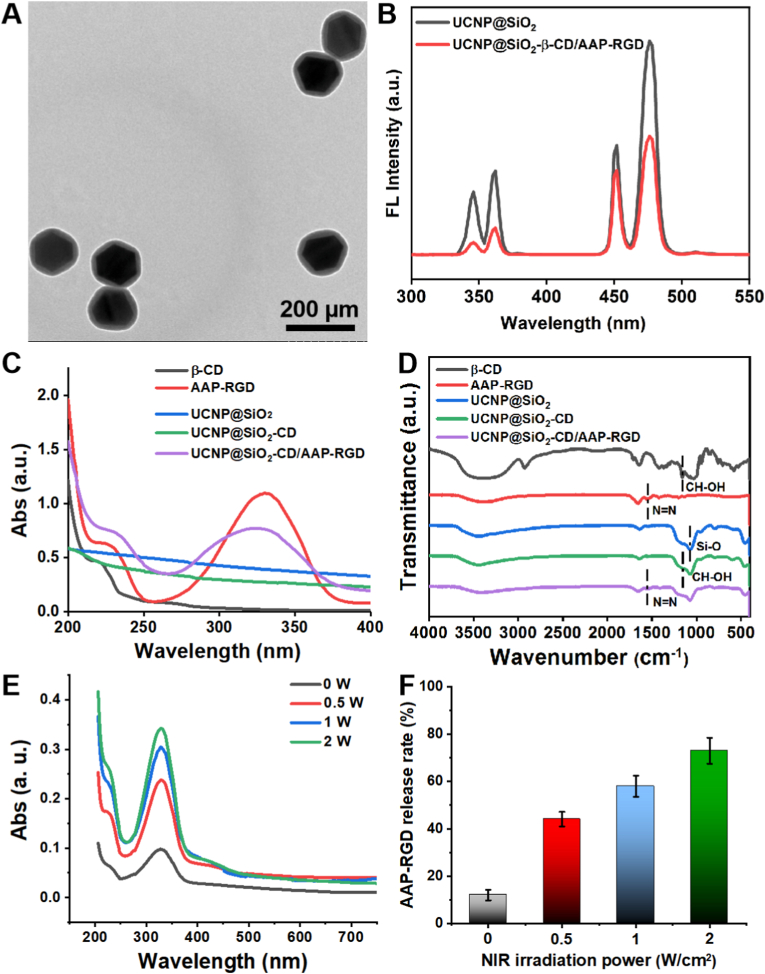
**Synthesis and Characterization of Functional UCNPs (UCNP@SiO_2_-CD/AAP-RGD).** (A) Representative TEM image of UCNP@SiO_2_-CD/AAP-RGD nanoparticles, revealing their uniform hexagonal morphology with an average diameter of 200 nm. (B) Fluorescence emission spectra of UCNP@SiO_2_-CD and UCNP@SiO_2_-CD/AAP-RGD under 808 nm excitation. The encapsulation of AAP-RGD led to a decrease in UV emission intensity. (C) UV–Vis absorption spectra of β-CD, AAP-RGD, UCNP@SiO_2_, UCNP@SiO_2_-CD, and UCNP@SiO_2_-CD/AAP-RGD, confirming successful synthesis steps. (D) FTIR spectra of β-CD, AAP-RGD, UCNP@SiO_2_, UCNP@SiO_2_-CD, and UCNP@SiO_2_-CD/AAP-RGD, providing further evidence of chemical conjugation. (E) Quantification of AAP-RGD release from UCNPs under 808 nm NIR irradiation (40 min) at varying power densities (0–2 W/cm^2^), demonstrating power-dependent release kinetics. (F) Release profile of AAP-RGD from UCNPs under different power of 808 nm NIR irradiation (0–2 W/cm^2^), achieving approximately 60 % detachment after 40 min irradiation with 1 W/cm^2^. Data represent mean ± s.e.m. (n = 3).

We first synthesized the functional UCNPs according to the procedures in Scheme 1A. First, OA-capped NaYF_4_:Yb/Tm UCNPs were synthesized and to avoid the quenching effect and enhance the fluorescence performance, a sensitizing layer (NaYF_4_:Yb/Nd) was coated onto the surface of OA-NaYF_4_:Yb/Tm via an epitaxial growth method. Fig. S1A shows that the Tm@Nd UCNPs (NaYF_4_:Yb/Tm@NaYF_4_:Yb/Nd) exhibit a dumbbell-like structure with the cross section of hexagonal. The cross-section diameter of the core-shell Tm@Nd UCNPs were determined, with an average size of 150 nm ± 3.5 nm and displayed a strong UV emission under 808 nm irradiation, which attributed to ^1^I_6_→^3^F_4_ (345 nm) and ^1^D_2_→^3^H_6_ (368 nm) transitions of Tm^3+^ ions doped. Afterward, the Tm@Nd UCNPs were encapsulated with silicon layer and then conjugated the cyclodextrin (SH-β-CD) by NHS-PEG-MAL linker to form the cyclodextrin modified UCNPs (UCNP@SiO_2_-CD). After assembling AAP-RGD onto UCNPs, the light-responsive functional UCNPs (UCNP@SiO_2_-CD/AAP-RGD) were investigated by TEM, fluorescence, UV–vis and FTIR. As shown in Fig. 1, TEM image indicated that the functional UCNPs have morphology and size and it possessed uniform hexagonal shapes with an average diameter of 200 nm (Fig. 1A). UCNP@SiO_2_-CD exhibited strong UV emission (Fig. 1B), capable of inducing conformational changes in AAP and modulating the host-guest interaction between AAP and β-CD, thereby triggering AAP-RGD separation from UCNPs to regulate cell behavior [22,23]. The observed decline in UV emission after AAP-RGD assembly (Fig. 1B) is attributed to an energy transfer or quenching effect between the UCNP and the surface-bound chromophore, providing further evidence of successful conjugation. The UV emission declined when AAP-RGD assembled to surface of UCNP@SiO_2_-CD and the UV–vis and FTIR results demonstrated the UCNP@SiO_2_-CD/AAP-RGD was prepared successfully (Fig. 1C and D). Under the 808 nm irradiation, the AAP-RGD could be detached from UCNPs and measured by UV–vis. The released amount exhibited dependence on laser power (Fig. 1E) and irradiation time (Fig. S2), demonstrating the light-responsive nature of the host-guest interaction between AAP and β-CD. The UV–vis measurements demonstrated a power- (Fig. 1E) and time-dependent (Fig. S2) release profile of AAP-RGD from the UCNPs, confirming the reversible, light-responsive host-guest interaction between AAP and β-CD. This controlled release profile, achieving ∼60 % detachment at 1 W/cm^2^ within 40 min (Fig. 1F), establishes the foundation for spatiotemporally modulating RGD density at the cell-material interface.

**Fig. 2:**
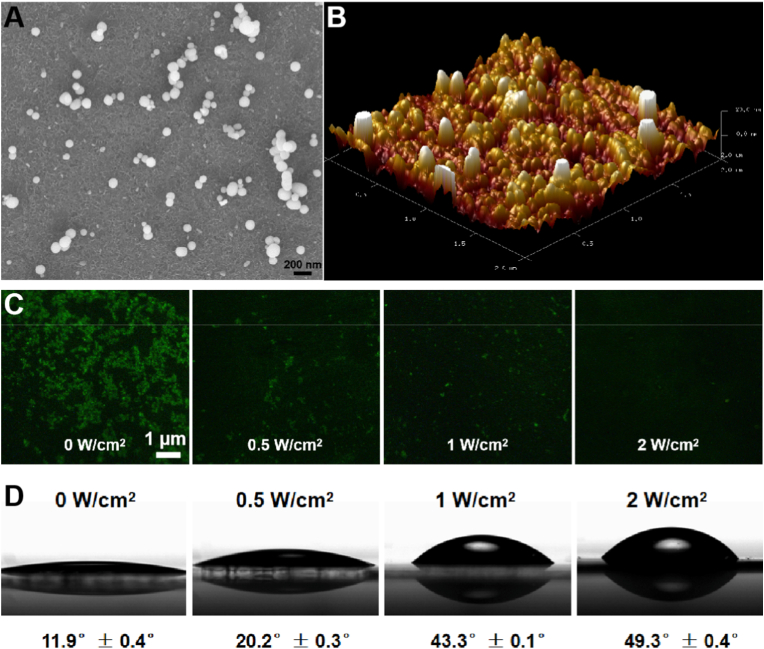
**Fabrication and Photoresponsive Properties of the UCNP-Substrate.** (A) SEM image of the UCNP-substrate, showing uniform distribution of functionalized UCNPs on the glass surface. (B) AFM image of the UCNP-substrate, corroborating surface topography and nanoparticle distribution. (C) Fluorescence microscopy images of FITC-labeled UCNP-substrate following exposure to 808 nm NIR irradiation at indicated power densities (0–2 W/cm^2^, 40 min). Decreasing FITC fluorescence intensity signifies NIR power-dependent detachment of AAP-RGD-FITC from the substrate surface. (D) Water contact angle measurements of the UCNP-substrate after exposure to varying NIR power densities (0–2 W/cm^2^, 40 min). Increasing contact angles (from 11.9° to 49.3°) reflect reduced surface hydrophilicity due to AAP-RGD detachment. Data represent mean ± s.e.m. (n = 3).

Next, we conjugated the functional UCNPs to glass plate by NHS-PEG-MAL linker to form the UCNP-substrate (Scheme 1B) [10,21]. The UCNP-substrate was characteristic by SEM (Fig. 2 A) and AFM (Fig. 2 B). From the results, the UCNPs were relatively uniformly distributed on the glass surface, with some minor, localized agglomerates observed, which helps with cell adhesion and growth. To explore whether an NIR laser could be used to dynamically control AAP-RGD detachment from UCNP-substrate, a fluorescence dye fluorescein isothiocyanate (FITC) was modified onto the peptide terminal of RGD via amino and carboxyl condensation. As shown in Fig. 2 C, after modification, the UCNP-substrate emitted bright green fluorescence. After the UCNP-substrate was treated with NIR laser with different power (from 0 to 2 W/cm^2^), the fluorescence intensity dropped slightly. Finally, the green fluorescence of UCNP-substrate decreased completely when upon exposed to high-intensity NIR irradiation (2 W/cm^2^), which indicating the control release of AAP-RGD from UCNP-substrate by adjusting NIR irradiation. Then, to test whether RGD peptide can influence the cell adhesion and spreading after activation, we conducted a water contact angle experiment (Fig. 2 D). The water contact angle increased from 11.9° to 49.3° as the NIR power increased from 0 to 2 W/cm^2^. This phenomenon showed the decreased substrate hydrophilicity due to the detachment of AAP-RGD with increasing NIR light intensity. Meanwhile, the shape of the water droplets tended to be an elliptical surface, indicating that the surface wettability and surface energy to the water were poor when the power was increased to 2 W/cm^2^, which could affect cell adhesion, spreading and multilineage differentiation [21]. The laser power range of 0–2 W/cm^2^ was systematically chosen as it was found to effectively span the dynamic range of RGD ligand release from the substrate surface (Fig. 1, Fig. 2C). This range allowed us to identify the critical power threshold (∼1 W/cm^2^) at which the MSC lineage commitment robustly switches from osteogenic to adipogenic. Importantly, this power density is well within the established ANSI safety limits for NIR exposure in biological tissues, underscoring the clinical translational potential of our platform.

### 2.2 Investigation of the cell adhesion and spread of MSCs on UCNP-substrate

**Fig. 3:**
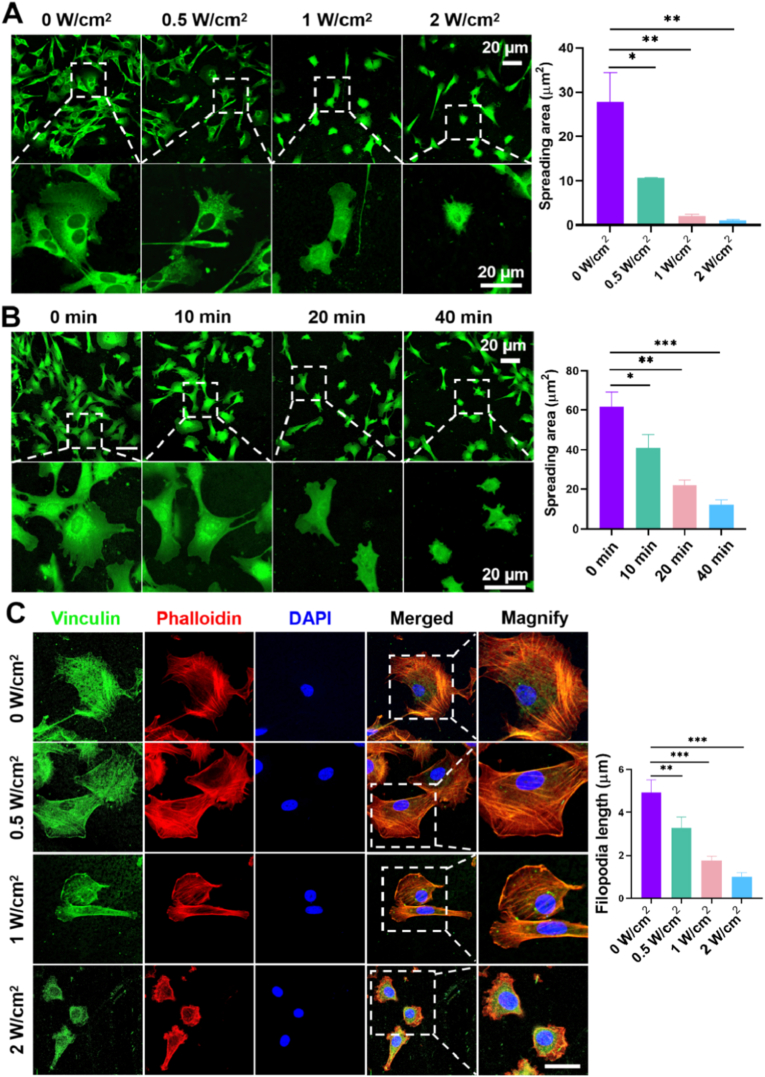
**NIR Spatiotemporal Control of MSC Adhesion and Spreading on the UCNP-Substrate.** (A) Representative phase-contrast images of MSCs cultured on UCNP-substrates pre-treated with 808 nm NIR irradiation at different power densities (0–2 W/cm^2^, 40 min) for 24 h. Increasing NIR power induced a morphological transition from well-spread (0 W/cm^2^) to rounded (2 W/cm^2^). (B) Representative phase-contrast images of MSCs cultured on UCNP-substrates pre-treated with 808 nm NIR irradiation (1 W/cm^2^) for varying durations (0–40 min) for 24 h. Prolonged irradiation time progressively reduced cell spreading. (C) Immunofluorescence staining of nuclei (DAPI, blue), vinculin (green), and F-actin (phalloidin, red) in MSCs cultured for 24 h on UCNP-substrates pre-treated with different NIR power densities (0–2 W/cm^2^, 40 min). High-power NIR irradiation (1–2 W/cm^2^) reduced vinculin-positive focal adhesion density and F-actin organization compared to low-power (0–0.5 W/cm^2^) or control conditions. Scale bar: 10 μm. Data represent mean ± s.e.m. (n = 3; ∗p < 0.05, ∗∗p < 0.01, ∗∗∗p < 0.001). (For interpretation of the references to color in this figure legend, the reader is referred to the Web version of this article.)

To study the cell adhesion and spreading of MSCs on UCNP-substrate, we cultured MSCs on UCNP-substrate after different power NIR irradiation processing. We first assessed the cytotoxicity of UCNP-substrate. As shown in Fig. S3, UCNP-substrate was first irradiated at different NIR powers and subsequently seeded with MSCs for 48 h. MSCs were stained using a live/dead cell viability assay (Annexin V/PI) and observed by fluorescence microscope. Results showed no obvious cytotoxicity of UCNP-substrate when MSCs were cultured on substrate after 48 h. Next, we investigated the cell adhesion and spreading of MSCs on UCNP-substrate after NIR irradiation processing (Fig. 3). We first used different power NIR light to irradiate UCNP-substrate and then cultured MSCs on substrate with 24 h, the MSCs could adhere on UCNP-substrate and present different cell morphology via different power NIR irradiation processed substrate (Fig. 3A). On UCNP-substrates without NIR irradiation (0 W/cm^2^), MSCs exhibited a spread morphology with a flat state, demonstrating more RGD forces act on the cell surface. We also established several control substrates, including RGD-modified substrate, UCNP-substrate (lacking RGD modification), and SiO_2_-RGD substrate, to investigate cell adhesion on different surfaces. As illustrated in Fig. S4, the RGD-modified substrate exhibited significant cell adhesion due to the adsorption effect of the RGD peptide. In contrast, the UCNP substrate demonstrated minimal cell adherence owing to the absence of RGD modification on its surface. The SiO_2_-RGD substrate similarly displayed substantial cell adhesion as a result of the presence of RGD modifications on its surface. Although cells adhered well to both the RGD-modified and SiO_2_-RGD substrates, variations in NIR light power did not influence cellular adhesion on these substrates since there were no UCNPs present to modulate the quantity of RGD available on their surfaces.

As laser power increased, MSCs spread gradually weakened, and the cell morphology gradually becomes smaller and rounder, which is caused by the gradually decreasing AAP-RGD via increasing NIR irradiation power. Additionally, we fixed the NIR irradiation power at 1 W/cm^2^ and varied the irradiation time from 0 to 40 min (Fig. 3B). Similarly, as the irradiation time increases, the cell adhesion and spread of MSCs on UCNP-substrate gradually weakens, and the cell morphology also shrinks and becomes round. Moreover, we used SEM to observe the cell adhesion and spread of MSCs on UCNP-substrate after different power NIR irradiation processing and the result showed that the cell adhesion and spread of MSCs on substrate could be adjusted by power of NIR light irradiation and it revealed 2.3-fold higher cellular contractility at 0.5 W/cm^2^ compared to 2 W/cm^2^ (Fig. S5). Thus, these results demonstrated that NIR light can be utilized to regulate the number of RGDs on UCNP-substrate, as well as the adhesion, spreading, and morphology of cells on substrate by adjusting NIR power and duration.

To further investigate the cell adhesion and spread of MSCs on UCNP-substrate, we performed immunofluorescence staining, as shown in Fig. 3C. Upon NIR irradiation of different power processing, MSCs were cultured on UCNP-substrate for 24 h and fixed for staining. Cells nuclear stained by DAPI, (Vinculin) focal contacts revealed by anti-vinculin antibody, (Actin) F-actin detected by TRITC-conjugated phalloidin and (Merge) merged stain of DAPI, phalloidin and vinculin. Immunofluorescence staining revealed enhanced efficacy of integrin β1 ligation and cell adhesion with higher density and focal adhesion with more spread morphology and less aspect ratio, including pronounced vinculin expression in FAs complexes on the 0 W/cm^2^ NIR irradiation processing, which possessed a high density of RGD ligands on the surface of UCNP-substrate in such a state. When we increased the power of NIR irradiation to process the UCNP-substrate, the upconverted UV light changed the conformation of AAP and triggered released AAP-RGD from substrate, which led to the reduction of RGD peptide quantity. The observed morphological transitions from spread (area = 610 ± 120 μm^2^) to rounded (area = 150 ± 35 μm^2^) correlated with vinculin-positive focal adhesion density, confirming the NIR light regulates cell morphology. This resulted in decreased cellular forces acting on the substrate surface and low fluorescent density of vinculin and F-actin for cell adhesion with less cell spread and morphology (2 W/cm^2^), thereby affecting the differentiation direction of MSCs (Scheme 1C).

In addition, the nucleus is the stiffest organelle in the cell and largely counterbalances the compressive forces imposed by actin cap stress fibers [24]. To verify whether the NIR irradiation processed UCNP-substrate could reorganize the nuclear morphology through the actin cap, the organization of the actin cap and the nuclear morphology were investigated thereafter. As shown in Fig. S6, MSCs on both the UCNP-substrates with low 0 and 0.5 W/cm^2^ NIR irradiation processing exhibited dense and aligned actin cap fibers with Lamin A/C staining, which is a main structural protein for nuclear envelope and mechanics and presented in the 3D organization of immunostaining to show the morphology of the nucleus [25]. Relatively, less and disordered actin cap fibers of MSCs were observed on high 1 and 2 W/cm^2^ NIR irradiation processed UCNP-substrate, which due to the lack of cellular contractility. Nucleus exhibited a smooth morphology on substrates irradiated at 0 and 0.5 W/cm^2^ NIR irradiation processed UCNP-substrate, whereas exhibited a less smooth and more irregular morphology and smaller nuclei were observed on those irradiated at 1 and 2 W/cm^2^ NIR irradiation processed UCNP-substrate, especially the 2 W/cm^2^ NIR irradiation processed substrate. Furthermore, the Lamin A/C expression of cells on the 2 W/cm^2^ NIR irradiation processed substrate was much lower than that on 0 W/cm^2^ NIR irradiation processed substrate. Lamin A/C serves as an indicator of nuclear stiffness and stability, and suppression of Lamin A/C would soften the nuclei and increase nuclear deformability, whereas overexpression of Lamin A/C could increase the nucleus's stiffness [26]. In our system, resulted in NIR light-controlled detachment of AAP-RGD on the substrate surfaces, reduced RGD peptide density potentially disrupting the force transmission and actin polymerization. Thus, the tension of the cellular actin cytoskeleton was greatly suppressed, which thereby increased the nuclear deformability on NIR irradiation processed substrate. In addition, it is important to consider the potential effect of the AAP-RGD released into the local environment upon NIR irradiation. However, these ligands are in a soluble form and at a low concentration, which is known to be far less effective at mediating stable focal adhesion formation and subsequent mechanotransductive signaling compared to high-density, substrate-tethered ligands. Therefore, the observed shifts in cell adhesion and lineage commitment are attributed to the dynamic reduction of immobilized RGD density on the substrate surface rather than a competitive effect from soluble RGD.

**Fig. 4:**
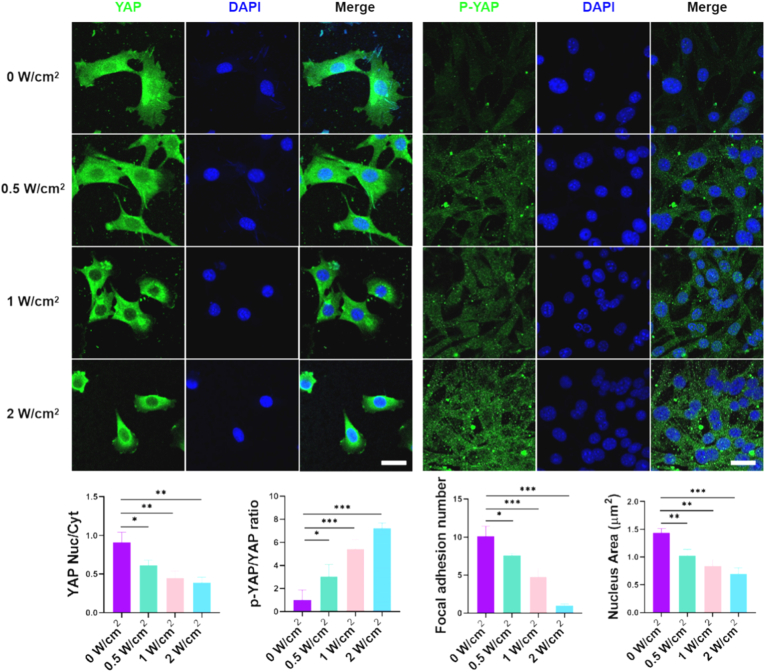
**NIR-Dependent Regulation of YAP/TAZ Subcellular Localization in MSCs**. Confocal microscopy images and corresponding quantification of the nuclear-to-cytoplasmic (Nuc/Cyt) ratio for YAP and P-YAP in MSCs cultured for 24 h on UCNP-substrates pre-treated with different 808 nm NIR power densities (0–2 W/cm^2^, 40 min). Low-power NIR irradiation (0–0.5 W/cm^2^) promoted nuclear localization of YAP. Increasing NIR power (1–2 W/cm^2^) shifted YAP predominantly to the cytoplasm and increased P-YAP expression. YAP and p-YAP images are representative fields from separate experimental replicates. Scale bar: 10 μm. Data represent mean ± s.e.m. (n = 3; ∗p < 0.05, ∗∗p < 0.01, ∗∗∗p < 0.001; N.S. = not significant).

Moreover, the transcriptional regulators YAP/TAZ have the ability to modulate the expression of numerous mechanosensitive genes [27]. The localization of YAP/TAZ within cells is influenced by cytoskeletal tension and nuclear mechanics [28]. Intracellular tension triggers the translocation of YAP/TAZ from the cytoplasm to the nucleus through the regulation of Ras-related GTPase RAP2, the ARID1A-containing SWI/SNF complex, and the opening of tension-stretched nuclear pores [29]. Typically, substrates exerting higher mechanical forces on cells promote increased nuclear localization of YAP/TAZ. We examined the subcellular distribution of YAP/TAZ using YAP immunostaining. As shown in Fig. 4, the results indicated that YAP predominantly localized in the nucleus when MSCs were cultured on a UCNP-substrate treated with 0 W/cm^2^ NIR irradiation. However, with an increase in the power of NIR irradiation, YAP became more concentrated in the cell cytoplasm, ultimately leading to the expression of YAP predominantly in the cytoplasm at 2 W/cm^2^ NIR irradiation. In addition, the expression of P-YAP also predominantly in the cytoplasm with an increase in the power of NIR irradiation, which suggests that cell transcriptional activity can be modulated synergistically by adjusting NIR levels. A potential confounding factor could be local heating induced by NIR irradiation. However, at the power densities used, we observed no significant temperature increase in the bulk medium, suggesting that photothermal effects are not the primary driver of the observed cellular responses. The clear dose-dependent correlation between NIR power, RGD release, and specific lineage commitment strongly supports a photochemically-driven mechanotransduction mechanism.

Consistent with the controlled RGD presentation, low NIR intensity (0, 0.5 W/cm^2^) robustly promoted focal adhesion assembly (evidenced by dense vinculin clustering, (Fig. 3C–S5), well-organized F-actin stress fibers, and the formation of a prominent actin cap (Fig. S6). This enhanced cytoskeletal tension translated into nuclear stiffening, characterized by smooth nuclear morphology and high Lamin A/C expression (Fig. S6), and drives nuclear translocation of the mechanotransducer YAP (Fig. 4). Conversely, high NIR intensity (1, 2 W/cm^2^) significantly suppressed these processes, leading to reduced vinculin and F-actin organization, disrupted actin cap, nuclear wrinkling/softening (low Lamin A/C), and consequent cytoplasmic retention of YAP (Fig. 3C–S6,S4). This coordinated response mirrors the established link between integrin ligation, cytoskeletal tension, nuclear mechanics, and YAP signaling observed in dynamic ECM remodeling [30], but here achieved through NIR spatiotemporal NIR photomodulation. Our findings strongly support the established paradigm that the YAP/TAZ signaling axis is a key mediator of mechanotransductive lineage commitment in MSCs [29]. The observed nuclear translocation of YAP under low NIR power (0–0.5 W/cm^2^) correlates directly with high cytoskeletal tension and osteogenic differentiation, consistent with reports where nuclear YAP activates osteogenic programs via transcription factors like RUNX2. Conversely, the cytoplasmic retention of YAP under high NIR power (1–2 W/cm^2^) is associated with relaxed cytoskeletal tension and the activation of adipogenic pathways, a process known to be mediated by factors such as PPARγ. While direct genetic manipulation of YAP was not performed in this study, the tight correlation between RGD density, cell mechanics, YAP localization, and lineage-specific gene expression provides compelling evidence for the central role of the YAP axis in our system.

It is important to acknowledge that cellular mechanotransduction is a highly complex process involving a multitude of interconnected pathways beyond the focal adhesion-YAP/TAZ axis investigated here. Notably, recent studies utilizing photo-tunable hydrogels have highlighted the cellular capability to sense rapid rigidity changes through the accumulation of mechanical signaling molecules, specifically involving the phosphorylation and dephosphorylation dynamics of Focal Adhesion Kinase (FAK) [31]. This finding aligns with our observation that dynamic alterations in ligand availability—analogous to rigidity changes—can rapidly trigger downstream signaling events. In our system, the specific integration of UCNP-triggered ligand release likely cooperates with these broader mechanosensing machineries, including the FAK axis and the Lamin A/C-mediated nuclear mechanotransduction we observed (Fig. S6), to dictate cell fate. Other contributing factors can include stretch-activated ion channels and alternative signaling pathways. Our platform provides a powerful tool to dissect these complex interactions by offering precise spatiotemporal control over the initial cell-matrix adhesion event, which serves as the primary trigger for this entire cascade of mechanobiological responses. To further investigate the change of cells spread morphology on UCNP-substrate, we cultured the MSCs on UCNP-substrate first and then used different power of NIR irradiation to process the substrate. After continuing culture the MSCs on UCNP-substrate for 24 h (Fig. S7), the confocal images showed that the spread morphology of MSCs has no remarkable change when the NIR irradiation was 0 W/cm^2^ and has obvious change from a flat and stretched state to a round cell when the NIR irradiation was 2 W/cm^2^, which lead to the detachment of AAP-RGD by upconverted UV from UCNPs. The adhesion and spreading behavior of MSCs on UCNP-substrate were dynamically regulated by NIR intensity. Subsequently, we investigated the multilineage differentiation outcomes under different irradiation.

The field of mechanobiology has seen remarkable advances in engineering biomaterial surfaces to control cell behavior. Recent elegant studies have demonstrated that precisely tuning adhesive ligand spacing [32] or ligand diffusibility [33] can exquisitely modulate focal adhesion dynamics and mechanotransduction. Our approach of on-demand ligand release complements these strategies by providing a distinct mechanism of control. While tuning spacing or mobility allows for a more analog regulation of receptor clustering, our method offers a robust, switch-like transition from a high-to a low-adhesion state. This is particularly advantageous for applications requiring a definitive and spatiotemporally precise switch in cell fate, thereby adding a unique and powerful tool to the repertoire for programming cellular responses.

**Fig. 5:**
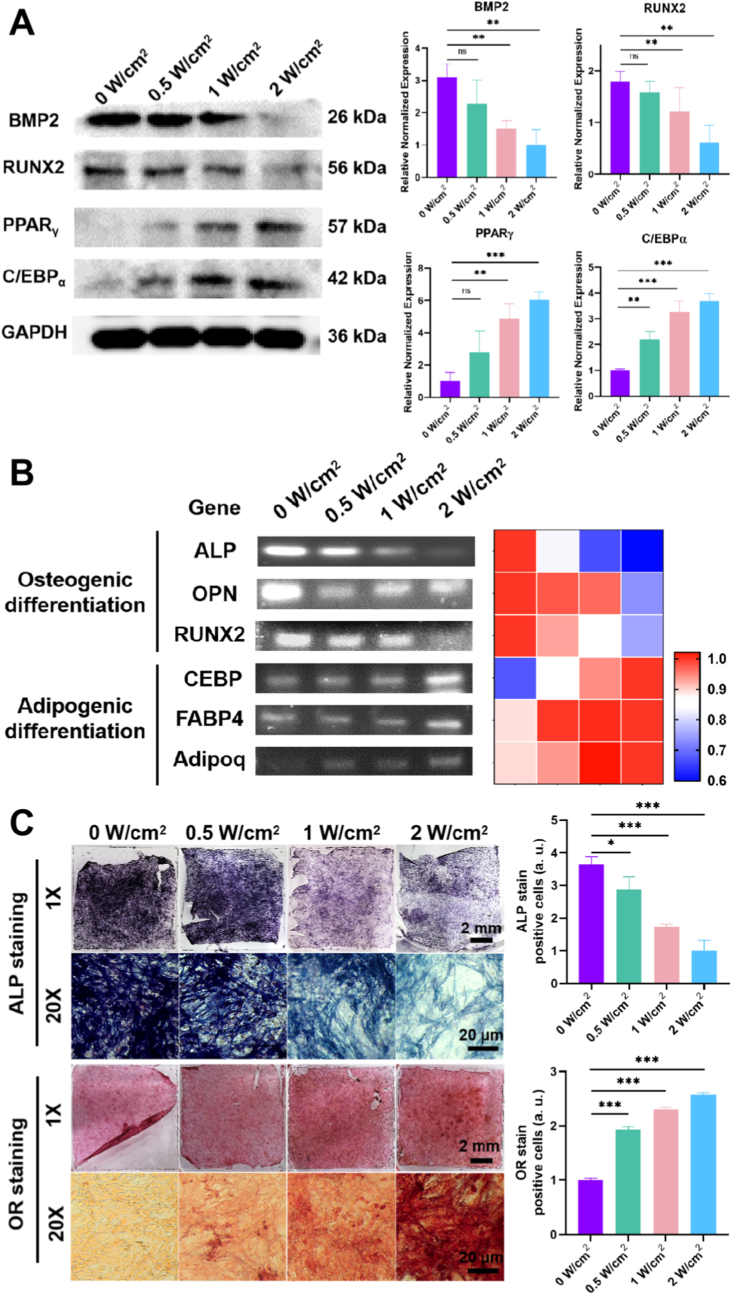
**NIR Spatiotemporal Control of MSC Multilineage Differentiation In Vitro**. (A) Western blot analysis of key osteogenic (BMP2, RUNX2) and adipogenic (PPARγ, C/EBPα) marker proteins in MSCs cultured on UCNP-substrates pre-treated with different NIR power densities (0–2 W/cm^2^, 40 min) and subsequently induced towards osteogenic/adipogenic lineages for 5 days. Low-power NIR (0–0.5 W/cm^2^) enhanced osteogenic marker expression, while high-power NIR (1–2 W/cm^2^) enhanced adipogenic marker expression. (B) Heatmap of RT-PCR analysis showing relative mRNA expression levels of osteogenic (ALP, OPN, RUNX2) and adipogenic (CEBPα, FABP4, Adipoq) marker genes in MSCs under the same conditions as in (A). Gene expression profiles corroborated protein data, confirming NIR power-dependent lineage commitment. (C) Representative images and quantification of Alkaline Phosphatase (ALP, purple) and Oil Red O (OR, red) staining in MSCs cultured on UCNP-substrates pre-treated with indicated NIR power densities (0, 0.5, 1, 2 W/cm^2^, 40 min) and differentiated for 7 days. Low-power NIR promoted intense ALP staining (osteogenesis), while high-power NIR promoted intense OR staining (adipogenesis). Data represent mean ± s.e.m. (n = 3; ∗p < 0.05, ∗∗p < 0.01, ∗∗∗p < 0.001; N.S. = not significant). (For interpretation of the references to color in this figure legend, the reader is referred to the Web version of this article.)

In addition, establishing a clear cause-and-effect relationship is paramount. While direct control experiments using substrates lacking either UCNPs or RGD ligands were not performed, a rigorous analysis of the system's components and the dose-dependent cellular responses provides a strong logical basis for our conclusions. i) The Essential Role of UCNPs: The photoisomerization of the arylazopyrazole (AAP) moiety, which drives its dissociation from β-cyclodextrin, requires high-energy UV photons (∼368 nm). The 808 nm NIR light used as the external trigger does not possess sufficient photon energy to induce this electronic transition directly. The sole function of the UCNPs in our system is to act as transducers, converting low-energy, tissue-penetrating NIR light into high-energy, localized UV light precisely at the substrate surface. Therefore, a control substrate with RGD but without UCNPs, when exposed to NIR, would be expected to show no response. The UCNP is not merely a component; it is the essential engine of the photo-response. ii) The Necessity of RGD Ligands: It is a fundamental principle of cell biology that MSCs require adhesion ligands like RGD for attachment, spreading, and subsequent mechanosensing. A control substrate coated with UCNPs but lacking the AAP-RGD ligand would be largely non-adhesive. Cells would fail to attach properly, precluding any meaningful analysis of differentiation. Thus, the baseline condition in our experiment—the complete system at 0 W/cm^2^ NIR—serves as the most relevant control, demonstrating the cellular response on the fully functionalized, high-adhesion substrate. iii) The Power of the Dose-Response: The most compelling evidence for our proposed causal link is the clear, graded, and power-dependent biological response that perfectly mirrors the graded, power-dependent release of the RGD ligand (compare Fig. 1F with Fig. 3, Fig. 4, Fig. 5). This internal dose-response relationship strongly refutes a non-specific thermal or light-based effect and serves as a powerful form of internal control, causally linking the number of surface ligands to the ultimate cell fate.

### 2.3 NIR spatiotemporal control of NIR mediated UCNP-substrate regulates multilineage differentiation of MSCs

**Fig. 6:**
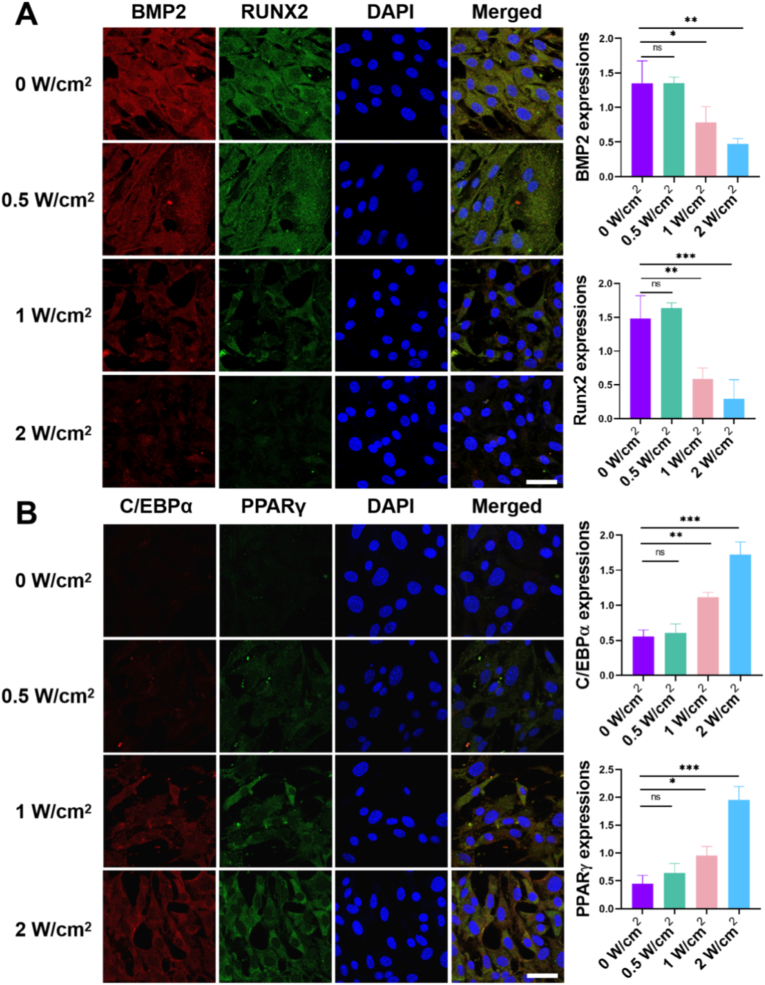
**Immunofluorescence Confirmation of NIR-Directed MSC Lineage Commitment In Vitro**. Immunofluorescent staining images of (A) osteogenic markers (BMP2, red; RUNX2, green) and (B) adipogenic markers (C/EBPα, green; PPARγ, red) in MSCs cultured on UCNP-substrates pre-treated with different NIR power densities (0–2 W/cm^2^, 40 min) and differentiated for 7 days. Fluorescence intensity quantification confirmed that low-power NIR irradiation enhanced osteogenic marker expression, while high-power NIR irradiation enhanced adipogenic marker expression. Scale bar: 20 μm. Data represent mean ± s.e.m. (n = 3; ∗p < 0.05, ∗∗p < 0.01, ∗∗∗p < 0.001; N.S. = not significant). (For interpretation of the references to color in this figure legend, the reader is referred to the Web version of this article.)

It is well-established that focal adhesion formation and cell spread morphology are key regulators of stem cell mechanotransduction and differentiation, processes essential for regenerative therapies [34]. We next evaluated whether modulated NIR irradiation could regulate MSCs multilineage differentiation in osteogenic/adipogenic induction media across four irradiation conditions including 0, 0.5, 1 and 2 W/cm^2^. Western blot analysis (Fig. 5A) revealed that low-intensity NIR irradiation (0 and 0.5 W/cm^2^) resulted in elevated expression of osteogenic proteins (BMP2 and RUNX2) but reduced expression of adipogenic proteins (PPARγ and C/EBPα) in MSCs, indicating significant osteogenic differentiation with this treatment condition. In contrast, under high-intensity NIR irradiation (1 and 2 W/cm^2^) there was a low protein expression of BMP2 and RUNX2 in MSCs but a high protein expression of PPARγ and C/EBPα, showing a significant adipogenic differentiation with this treatment condition. Additionally, RT-PCR analysis was conducted to further assess the cell differentiation-related gene expression in MSCs on the UCNP-substrates with different powers of NIR irradiation. As shown in Fig. 5B, results of RT-PCR heat-map analysis demonstrated that compared with high-power NIR treatment substrate (1 and 2 W/cm^2^), the osteogenic differentiation related genes (ALP, OPN, RUNX2) in low-power NIR treatment substrate (0 and 0.5 W/cm^2^) were substantially upregulated while the adipogenic differentiation related genes (CEBP, FABP4, Adipoq) were downregulated from MSCs. And the high-power NIR treatment substrate has a high gene expression of CEBP, FABP4, Adipoq, which indicated a significant adipogenic differentiation of MSCs on these treatment substrate. Furthermore, alkaline phosphatase (ALP) and oil red O (OR) staining were performed to further investigate the multilineage differentiation of MSCs. As shown in Fig. 5C, the MSCs cultured on the low-power NIR treatment substrate showed a deep ALP stain (dark purple) and a light OR stain, and MSCs cultured on high-power irradiated substrates exhibited faint ALP staining but intense OR staining, indicating a propensity for adipogenic differentiation. Conversely, MSCs on low-power irradiated substrates showed the opposite trend, favoring osteogenic differentiation. Finally, subsequent immunofluorescence staining further corroborated the gene expression results. As shown in Fig. 6A, the expression of BMP2/RUNX2 (osteogenic differentiation polarized marker) was the most remarkable in the 0 W/cm^2^ group, and that decreased from the 0.5 W/cm^2^ to the 1 W/cm^2^ groups, with the lowest expression observed in the 2 W/cm^2^ group. The expression of C/EBPα/PPARγ (adipogenic differentiation polarized marker), however, exhibited an exactly reverse trend, with the lowest expression observed in the 0 W/cm^2^ group but the highest expression observed in the 2 W/cm^2^ group (Fig. 6B). Strikingly, the NIR-controlled RGD density directed MSC lineage commitment along distinct pathways. Under low-intensity NIR (0, 0.5 W/cm^2^), which promotes high cytoskeletal tension and nuclear YAP, MSCs exhibited robust osteogenic differentiation, as evidenced by significantly upregulated expression of osteogenic markers BMP2, RUNX2, ALP, and OPN (2–3 fold increase vs. high intensity, Fig. 5, Fig. 6). Conversely, high-intensity NIR (1, 2 W/cm^2^), associated with reduced tension and cytoplasmic YAP retention, preferentially induced adipogenic differentiation, marked by elevated levels of PPARγ, C/EBPα, FABP4, and Adipoq (4–5 fold increase vs. low intensity, Fig. 5, Fig. 6). Critically, the differentiation threshold (e.g., 1 W/cm^2^) corresponds to clinically safe NIR exposure limits, underscoring its translational potential. This power-dependent steering of differentiation is mechanistically linked to the YAP-mediated mechanotransduction pathway: Nuclear YAP at low power activates osteogenic programs (e.g., via RUNX2), while cytoplasmic YAP at high power enables adipogenic commitment (e.g., via PPARγ) [35]. Furthermore, temporal control was demonstrated through switching NIR intensity at defined time points, effectively redirecting differentiation outcomes and offering unprecedented spatiotemporal precision in programming stem cell fate NIR spatiotemporally.

Moreover, we established several control substrates, including an RGD-modified substrate, a UCNP substrate (lacking RGD modification), and a SiO_2_-RGD substrate, to investigate cell differentiation on various surfaces. As illustrated in Fig. S8 (ALP staining) and Fig. S9 (Oil Red O staining), the RGD-modified substrate exhibited significant ALP staining due to the adsorption effect of the RGD peptide. In contrast, the UCNP substrate demonstrated minimal ALP staining owing to the absence of RGD modification on its surface. The SiO_2_-RGD substrate similarly displayed substantial ALP staining as a result of the presence of RGD modifications. Although cells showed considerable ALP staining on both the RGD-modified and SiO2-RGD substrates, variations in NIR light power did not influence ALP staining on these substrates since there were no UCNPs present to modulate the quantity of available RGD on their surfaces. In Fig. S9, Oil Red O staining was insignificant across all control groups because the adhesion force of RGD caused cells to adopt a relatively flat morphology on the substrates. Consequently, MSCs were more inclined to differentiate into osteoblasts rather than adipocytes.

Thus, these findings collectively suggest the observed NIR-dependent modulation of MSC adhesion and cytoskeletal tension (Fig. 3) directly translated into divergent mechanotransductive signaling. Specifically, low-intensity NIR (0–0.5 W/cm^2^) reinforced focal adhesion assembly and actin cap formation, driving nuclear accumulation of YAP (Fig. 4) — a master regulator of mechanosensitive transcription. Conversely, high-intensity irradiation (1–2 W/cm^2^) suppressed cytoskeletal contractility, resulting in cytoplasmic YAP retention and nuclear softening (Fig. 4, S7). This tension-to-YAP axis ultimately dictated lineage commitment: nuclear YAP at low power promoted osteogenesis via BMP2/RUNX2 upregulation, while cytoplasmic YAP at high power activated PPARγ-mediated adipogenesis (Fig. 5, Fig. 6). Quantitative correlation analysis revealed a strong positive relationship between nuclear YAP/TAZ ratio and osteogenic marker expression (R^2^ = 0.89, *p* < 0.001, not show), further validating the mechanochemical basis of differentiation steering. These results align with the established paradigm of stiffness-dependent differentiation [29], yet uniquely demonstrate photochemical control over the mechanobiological cascade through dynamic RGD-integrin engagement. And our findings extend the conventional stiffness-YAP-differentiation paradigm by demonstrating that light-tunable RGD density can dynamically reconfigure cytoskeletal tension, thereby offering spatiotemporal control over stem cell fate.

### 2.4 In vivo regulation of cell adhesion and multilineage differentiation of MSCs via NIR mediated UCNP-substrate

**Fig. 7:**
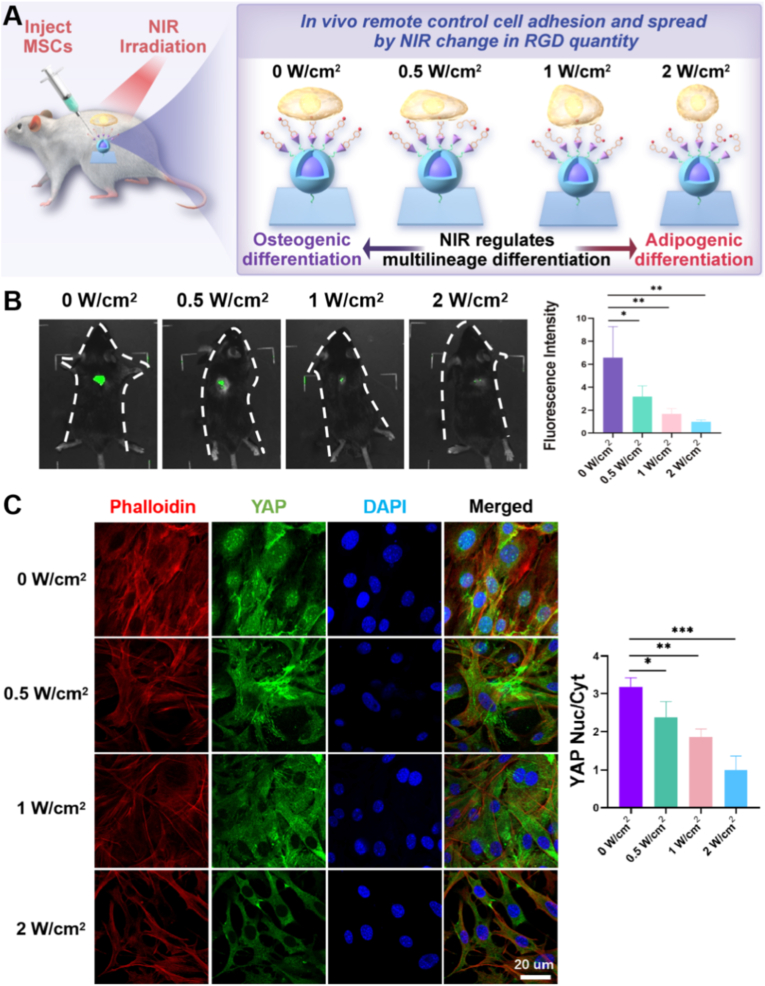
**In Vivo NIR Spatiotemporal Control of MSC Adhesion, Spreading, and YAP Signaling**. (A) Schematic illustration of the in vivo experimental setup: subcutaneous implantation of the UCNP-substrate in mice, injection of MSCs, and localized NIR irradiation to dynamically control RGD density and subsequent cell behavior. (B) In vivo fluorescence imaging (FITC channel) and corresponding signal quantification at the implantation site following 808 nm NIR irradiation at varying power densities (0–2 W/cm^2^, 40 min). Decreasing FITC fluorescence intensity confirmed NIR power-dependent AAP-RGD-FITC detachment from the implanted substrate. (C) Immunofluorescence staining of F-actin (phalloidin, red), YAP (green), and nuclei (DAPI, blue) in MSCs recruited to the implanted UCNP-substrate under different NIR power irradiation. Quantification of the nuclear/cytoplasmic (Nuc/Cyt) ratio of YAP confirmed that low-power NIR (0–0.5 W/cm^2^) promoted nuclear YAP localization, while high-power NIR (1–2 W/cm^2^) induced cytoplasmic retention. Scale bar: 20 μm. Data represent mean ± s.e.m. (n = 3; ∗p < 0.05, ∗∗p < 0.01, ∗∗∗p < 0.001). (For interpretation of the references to color in this figure legend, the reader is referred to the Web version of this article.)

UCNP-based nanocomposites offer distinct advantages for in vivo applications, including deep tissue light penetration and precise temporal control [36]. Therefore, we next examined whether the UCNP-substrate could regulate cell adhesion and control multilineage differentiation of MSCs effectively by NIR light in vivo. For in vivo experiments, UCNP-substrates were implanted subcutaneously in the backs of mice and then exposed with NIR in different situations (Fig. 7A). We first assessed NIR-triggered AAP-RGD detachment in vivo, and the result was shown in Fig. 7B. The release profile of AAP-RGD-FITC in vivo mirrored that observed in vitro. With the increase of NIR intensity, the green fluorescence intensity in the backs of mice gradually decreased, indicating that AAP-RGD was gradually released. Next, we verified whether NIR could control the UCNP-substrate to detach the adhesive RGD peptide (AAP-RGD) and regulate cell adhesion in vivo. We implanted the UCNP-substrate in the back of mice and then used NIR to irradiate the back of mice and injected MSCs to the back of mice. After 5 days feeding, we took out the UCNP-substrate from the back of mice and stained the substrate with immunofluorescence (IF) for YAP, which is the mechanosensitive transcriptional activator [37]. As shown in Fig. 7C, immunofluorescence staining confirmed that low-power NIR irradiation (0 and 0.5 W/cm^2^) stimulated significantly higher adherent cell density and focal adhesion of MSCs over a wider area and YAP expression in nucleus. With the extension of the period and the increase of the NIR light intensity in high-power NIR irradiation regulated switching (1 and 2 W/cm^2^), the expression of YAP gradually decreases in the nucleus and increases in the cytoplasm, which lead to the detachment of AAP-RGD on substrate by adjusting NIR irradiation. These results indicated that the UCNP-substrate could efficiently regulate cell adhesion and spreading in vivo through NIR-controlled release of AAP-RGD.

To verify the origin of substrate-adherent cells in vivo, MSCs were first labeled in vitro with the red fluorescent membrane dye PKH26. Successful incorporation of the dye was confirmed by fluorescence microscopy (Supplementary Fig. S10). These pre-labeled MSCs were then detached, collected, and administered via subcutaneous injection at the substrate implantation site. Following a designated implantation period, the substrates were harvested, rinsed thoroughly (e.g., with PBS) to remove non-adherent cells, and subsequently analyzed by fluorescence microscopy. The detection of distinct PKH26-positive (red fluorescent) cells adhering to the harvested substrates confirmed their identity as the originally injected MSC population.

**Fig. 8:**
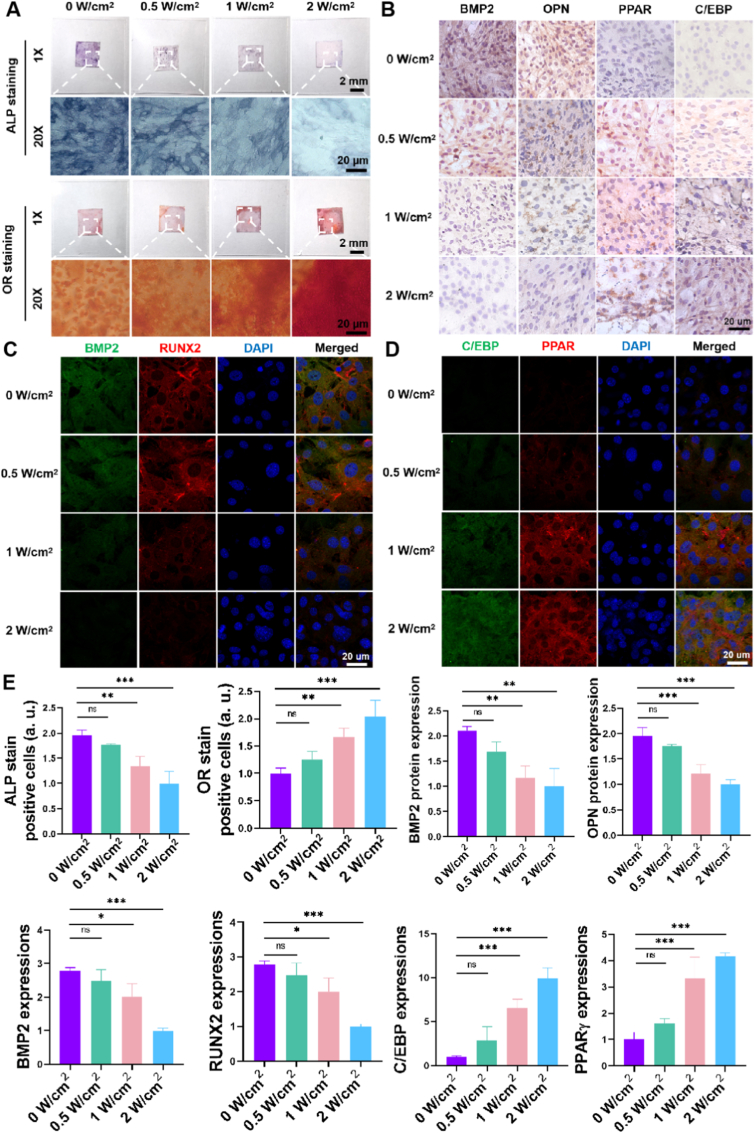
**In Vivo Validation of NIR-Spatiotemporal Control of MSC Multilineage Differentiation**. (A) Representative images and quantification of Alkaline Phosphatase (ALP, purple) and Oil Red O (OR, red) staining on explanted UCNP-substrates after subcutaneous implantation, MSC injection, and exposure to different NIR power densities (0–2 W/cm^2^) during a 7-day differentiation period in vivo. Results recapitulated in vitro findings: low-power NIR favored osteogenesis (strong ALP), high-power NIR favored adipogenesis (strong OR). (B) Immunohistochemistry (IHC) staining for osteogenic (RUNX2, OPN) and adipogenic (PPARγ, C/EBPα) marker proteins on explanted substrates. Low-power NIR irradiation enhanced osteogenic marker expression, while high-power NIR irradiation enhanced adipogenic marker expression. (C, D) Immunofluorescence staining of explanted substrates for (C) osteogenic markers (BMP-2, Cy5-red; RUNX2, FITC-green) and (D) adipogenic markers (PPARγ, Cy5-red; C/EBPα, FITC-green), confirming NIR power-dependent lineage specification in vivo. (E) Statistical quantification of positive staining areas for ALP/OR, IHC markers (RUNX2, OPN, PPARγ, C/EBPα), and immunofluorescence markers (BMP2, RUNX2, PPARγ, C/EBPα) across different NIR treatment groups, demonstrating significant differences in osteogenic vs. adipogenic differentiation outcomes. Data represent mean ± s.e.m. (n = 3; ∗p < 0.05, ∗∗p < 0.01, ∗∗∗p < 0.001; N.S. = not significant). (For interpretation of the references to color in this figure legend, the reader is referred to the Web version of this article.)

Next, we evaluated multilineage differentiation of MSCs on UCNP-substrate upon NIR irradiation adjustment in vivo. The UCNP-substrate was implanted into the back of mice first and then with different power NIR irradiation. Next, we injected the MSCs into the back of mice and took out the UCNP-substrate from the back of mice 7 days later. The extracted substrates were performed ALP/OR, immunohistochemistry, and immunofluorescence staining. As shown in Fig. 8A, results of ALP/OR staining on UCNP-substrates in vivo recapitulated the findings from in vitro studies. The MSCs on the substrate with low-power NIR irradiation tended to differentiate into osteoblasts, and the MSCs on substrate with high-power NIR irradiation tended to differentiate into adipocytes. Next, the immunohistochemistry stain was performed to further investigate the multilineage differentiation of MSCs on the UCNP-substrate with different NIR power treatments in vivo. As shown in Fig. 8B, the BMP-2 and OPN proteins showed a dark stain with a high expression, which indicated a significant osteogenic differentiation of MSCs by the low-power NIR treatment (0 and 0.5 W/cm^2^). In contrast, when the substrate was treated with the high-power NIR (1 and 2 W/cm^2^), the PPARγ and C/EBPα proteins showed a dark stain with a high expression, which indicated a significant adipogenic differentiation of MSCs.

In addition, the immunofluorescence stain was carried out to verify further the multilineage differentiation of MSCs on UCNP-substrate (Fig. 8C and D). The immunofluorescence staining revealed a stronger signal of the cy5-conjugated antibody against BMP-2, and a strong green signal of the FITC-conjugated antibody against RUNX2 was found in the cytoplasm of MSCs (Fig. 8C), indicating the emergence of osteogenic differentiation in MSCs with low-power NIR. In addition, a strong red signal of the cy5-conjugated antibody against PPARγ and a strong green signal of the FITC-conjugated antibody against C/EBPα were found in the cytoplasm of MSCs (Fig. 8D), which suggested a significant adipogenic differentiation of MSCs that were cultured on a UCNP-substrate exposed to high-power NIR. Statistical analysis confirmed that the results of ALP/OR staining, immunohistochemistry, and immunofluorescence staining for cells undergoing multilineage differentiation (osteogenic/adipogenic) differed significantly across treatment groups (p < 0.05, Fig. 8E). Unlike approaches requiring invasive injection of chemical triggers (e.g., EDTA/BP) [11] or cytotoxic UV light [38], our platform leverages deep-penetrating, biocompatible NIR light to achieve non-invasive, spatiotemporal, and reversible control over stem cell adhesion and multilineage differentiation within clinically safe exposure limits (e.g., 1 W/cm^2^). It should be noted that the images of cells from the in vivo experiments (Fig. 7, Fig. 8) were taken from the substrates after they were explanted and washed. A thin, translucent fibrous capsule, indicative of a mild foreign body response, was observed around the implants upon retrieval. However, MSCs were clearly able to adhere to and differentiate on the substrate surface, allowing for direct analysis of cell behavior on the material itself. This capability to NIR spatiotemporally program stem cell fate in situ holds immense promise for applications like localized osteogenesis/adipogenesis regulation in osteoporosis treatment.

**Fig. 9:**
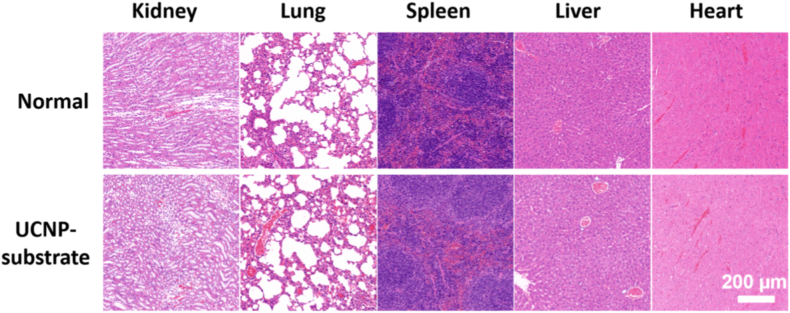
**HE Staining of the Heart, Liver, Spleen, Lung and Kidney of Mice with or without UCNP-Substrate Implantation.** The in vivo implantation of UCNP-substrate did not show obvious toxicity for the mice. Normal: on implantation.

Finally, we evaluated the in vivo toxicity of the UCNP-substrate by implanting it into the dorsal regions of mice. After two weeks, the substrates were removed, and the animals were euthanized to harvest the heart, liver, spleen, lung, and kidney tissues. These tissues were subsequently fixed, embedded, sectioned, and subjected to hematoxylin and eosin (H&E) staining. As shown in Fig. 9, no significant histopathological abnormalities were observed, indicating that UCNP-substrate implantation does not induce notable toxic effects in mice, thus demonstrating its biocompatibility under the tested conditions.

The use of lanthanide-doped UCNPs in biomedical applications necessitates careful consideration of their long-term biocompatibility. A key feature of our design is the encapsulation of the rare-earth-doped core within a dense and stable silica (SiO_2_) shell. This silica coating is a well-established strategy to enhance biocompatibility and, crucially, to prevent the leaching of potentially toxic lanthanide ions into the biological environment. Numerous studies have demonstrated that with proper surface engineering, such as the silica coating employed here, UCNPs exhibit good biocompatibility and low cytotoxicity for in vivo applications, making them a promising platform for translatable regenerative therapies [17].

On the other hand, the significance of the dynamic tunability of the extracellular matrix revealed by our work for cell fate determination also coincides with the recent research on the role of cellular mechanical sensing in histogenesis and disease occurrence [39]. Our research not only resolves the design issues of specific biomaterials, but more importantly, it provides a “tool" or “platform" that opens up new possibilities for a deeper understanding of how cells sense and respond to dynamic mechanical signals, as well as how to utilize these signals for intervention.

## 3 Conclusion

In summary, we have developed a NIR-light-responsive upconversion platform integrated with a host-guest photochemical system, enabling spatiotemporal, non-invasive control over MSC fate through phototunable RGD-integrin interactions. This system uniquely decouples photochemical stimulation enabling dynamic regulation of cell adhesion, cytoskeletal organization, nuclear mechanics, YAP/TAZ signaling, and ultimately, lineage commitment (osteogenesis vs. adipogenesis). Critically, in vivo validation confirmed the platform's capability to NIR spatiotemporally guide differentiation within clinically safe NIR power limits (e.g., 1 W/cm^2^), operating non-invasively without chemical injections and circumventing the penetration and toxicity limitations associated with UV light. The modular design readily accommodates the integration of additional photoresponsive factors (e.g., VEGF, neurotrophins), thereby opening avenues for engineering complex multicellular tissues. By synergizing material innovation with mechanobiological principles, this platform offers a translatable strategy for the design of intelligent biomaterials for personalized regenerative medicine, such as spatiotemporal regulation of the osteogenic/adipogenic balance in osteoporosis. While the individual signaling components, such as integrin-mediated adhesion and the YAP/TAZ mechanotransduction cascade, are well-understood, the novelty of this work lies in the development of an advanced opto-material platform that provides an external, non-invasive handle to precisely control this complex cellular machinery. Our objective was not to uncover new biological pathways, but rather to engineer a sophisticated tool that can hijack these known pathways for therapeutic ends. By demonstrating that the intensity of biocompatible NIR light can be used to quantitatively steer MSC lineage commitment in vivo, we provide a proof-of-concept for a new class of ‘smart’ biomaterials capable of directing tissue regeneration with unprecedented spatiotemporal precision.

## 4 Materials and method

### 4.1 Materials

All reagents were purchased from Aladdin (Shanghai, China). Arylazopyrazole (AAP) was purchased from Beijing Famous Pharmaceutical Technology Co., Ltd (China). AAP-RGD was synthesized by Top-peptide Co. Ltd (Shanghai, China). Phosphae-buffered saline (PBS), fetal bovine serum (FBS), DMEM medium were purchased from Gibco (USA). And penicillin/streptomycin were purchased from Solarbio (Beijing, China). PCR primers and reagents were purchased from Sangon (Shanghai, China). Antibodies were purchased from Affinity Biosciences (Cincinnati, OH, USA). The 808 nm-NIR laser was from Lasever Inc. (Ningbo, China). Transmission electron microscopy (TEM) was performed on JEM-1400 plus (JEOL, Japan) with an accelerating voltage of 120 kV. Scanning electron microscope (SEM) was performed on ZEISS Ultra 55 (Germany). UV–vis absorption spectra were recorded on DU800 UV–visible spectrophotometer (USA). Fourier transform infrared spectrometry (FTIR) was performed on a Nicolet iS50 FTIR (Thermo Scientific, USA). The cell images were collected by NIB 900 inverted fluorescence microscope (Nexcope, Shanghai, China) and confocal laser scanning microscopy (CLSM, Zeiss LSM 880, Germany). The RT-PCR was performed on LifeECO PCR instrument (BIOER, Hangzhou, China). The western blot system was a Bio-Rad mini WB system (Bio-Rad, Hercules, CA, USA). RT-PCR and Western blot results were determined by fluorescence and chemiluminescence imaging system (ChemiScope 6100, Clinx, Shanghai, China), the animal imaging system was also from Clinx (ChemiScope IVScope 8000pro, Shanghai, China). Female C57BL/6 mice were purchased from Guangdong medical laboratory center (Guangzhou, China).

### 4.2 Synthesis of UCNPs (NaYF_4_:Yb/Tm@NaYF_4_:Yb/Nd) and UCNP@SiO_2_-NH_2_

Using a conventional UCNP synthesis method, add 15 mL of oleic acid and 30 mL of 1-octadecene into a 100-mL flask (all reagents were purchased from Aladdin). Weigh 1.59 mmol of YCl_3_∙6H_2_O, 0.4 mmol of YbCl_3_∙6H_2_O, and 0.01 mmol of TmCl_3_∙6H_2_O, then add these to the flask and disperse them ultrasonically. Heat the solution slowly to 160 °C and maintain this temperature for 30 min until a uniform yellow solution is obtained. Cool downs to room temperature.

Simultaneously, weigh 5 mmol of NaOH and 8 mmol of NH_4_F, dissolve them in 10 mL of methanol, and stir for 30 min to achieve uniform dispersion. Add this methanol solution to the oleic acid mixture. Stir the combined mixture at 50 °C for 40 min, then gradually heat it to 300 °C under an argon atmosphere and maintain this temperature for 1 h. Cool the mixture to room temperature. Precipitate the products by adding 10 mL of ethanol, then collect them via centrifugation at 8000 rpm for 5 min. Wash the precipitate three times with ethanol and cyclohexane, each wash lasting 5 min at 8000 rpm. Dry the UCNPs naturally and store them at 4 °C. The synthesis of NaYF_4_:Yb/Tm@NaYF_4_:Yb/Nd is similar to the synthetic process of NaYF_4_:Yb/Tm, except that cyclohexane-soluble NaYF_4_:Yb/Tm is added with NaOH and NH_4_F.

Next, reverse microemulsion method was used to synthesize the UCNP@SiO_2._ Briefly, Dissolve 40 mg of UCNPs in 8 mL of cyclohexane, then sonicate for 30 min and stir for an additional 30 min to ensure uniform dispersion. Add 1 mL of hexane, 1 mL of Triton X-100, 0.2 mL of deionized water, and 0.1 mL of NH_3_∙H_2_O to the solution. Stir the mixture at room temperature for 2 h. Then add 50 μL of TEOS and stir for 12 h. Finally, introduce 5 μL of APTES, stir for 6 h, and then add 5 mL of acetone to break the emulsion. Wash the products with ethanol three times and dry at room temperature for 12 h to obtain UCNP@SiO_2_-NH_2_.

### 4.3 Synthesis of UCNP@SiO_2_-CD

UCNP@SiO_2_-NH_2_ (40 mg) were dissolved in 10 mL of DMF, and NHS-PEG-MAL (1 mg) was added to the solution. The mixture was stirred for 24 h to form the UCNP@SiO_2_-PEG-MAL with the reaction between NH_2_ and NHS. The resulting products were collected by centrifugation and washed three times with absolute ethanol. The UCNP@SiO_2_-PEG-MAL were then re-dissolved in 10 mL of DMF containing SH-β-CD (1 mg) to stir for 24 h at RT. During this process, the thiol groups on SH-β-CD reacted with the maleimide groups introduced by PEG via a thiol–maleimide Michael addition under mild conditions to form the UCNP@SiO_2_-PEG-β-CD. Finally, the reactive solution was centrifuged and washed with ethanol to collect the UCNP@SiO_2_-PEG-β-CD (UCNP@SiO_2_-CD).

### 4.4 Synthesis of functionalized UCNPs (UCNP@SiO_2_-CD/AAP-RGD)

UCNP@SiO_2_-CD (5 mg) was added to a 1:1 mixture of DMSO and ultrapure water, and thoroughly mixed. AAP-RGD was then added, and the solution was stirred for 24 h to form the UCNP@SiO_2_-CD/AAP-RGD through supramolecular self-assembly between cyclodextrin and azobenzene. The “release rate" is the amount released by NIR, expressed as a percentage of this total loaded amount. Therefore, the release rate = the amount of AAP-RGD measured in the supernatant solution after NIR irradiation and centrifugation/the amount of AAP-RGD bound to cyclodextrin of UCNPs; and the amount of AAP-RGD bound to cyclodextrin of UCNPs = the total amount of AAP-RGD added for host-guest reaction with UCNPs - the amount of AAP-RGD detected by UV–vis in the supernatant after host-guest reaction and centrifugation. After centrifugation, functionalized UCNPs were obtained and stored at 4 °C.

### 4.5 Conjugation of functionalized UCNPs to cover glass to form UCNP-substrate

Cover glass (22 × 22 mm) was washed with DI water and then immersed in piranha solution (98 % H_2_SO_4_/30 % H_2_O_2_ = 3:1 v/v) for 6 h to remove surface impurities and activate the hydroxyl groups. The piranha solution was recovered for recycling three times. The cover glass was then cleaned with DI water and absolute ethanol. MPTMS (0.01 % v/v) and DIPEA (0.5 % v/v) were prepared for the subsequent experiment. The cover glass was sequentially immersed in MPTMS (0.01 % v/v) and DIPEA (0.5 % v/v) containing UCNP@SiO_2_-CD/AAP-RGD (1 mg) and shaken for 12 h to obtain the UCNP-substrate with the reaction between -SH on glass and MAL on surface of UCNPs. The UCNP-substrate was then washed three times with DI water and ethanol to remove the unconnected UCNPs.

### 4.6 Coupling of fluorescein isothiocyanate (FITC) to the UCNP-substrate

After synthesizing the UCNP-substrate, it was placed into a 6-well plate containing 1 mL of DMF. FITC-NH_2_ (1 mg) was added, along with EDC and NHS, and the mixture was shaken at room temperature for 12 h. The FITC-NH_2_ was connected on UCNP-substrate with the reaction of -NH_2_ and -COOH of RGD. Following the reaction, the UCNP-substrate-FITC was washed three times with ethanol and then dried. The final product was stored at 4 °C in the dark.

### 4.7 NIR-triggered release of AAP-RGD from functionlized UCNPs and UCNP-substrate

1 mL of UCNP@SiO_2_-CD/AAP-RGD (1 mg/mL in PBS) was exposed to 808 nm NIR light at various power levels (0, 0.5, 1, 2 W/cm^2^, 40 min) and different duration time (0, 20, 40, 60, 80 min, 1 W/cm^2^) at room temperature, under dark conditions. After irradiation, the solution was centrifuged to collect the supernatant and test the UV absorption of AAP-RGD for measuring the NIR-triggered release of AAP-RGD. Similar operations for UCNP-substrate. For contact angle experiment, the UCNP-substrates were irradiated with NIR light and then tested the contact angle of water drop with different irradiation condition. For FITC release experiment, the UCNP-substrates were observed with fluorescent microscope after different condition NIR irradiation.

### 4.8 Cell culture

Mesenchymal stem cells (MSCs) derived from mice were purchased from Cyagen Biosciences Inc. (Guangzhou, China). The cells were seeded in 6-well plates containing UCNP-substrates at a density of 10,000 cells per well. They were cultured in DMEM supplemented with 10 % fetal bovine serum (FBS), 1 % penicillin-streptomycin, and 1 % glutamine, at 37 °C in a humidified atmosphere with 5 % CO_2_. Prior to cell experiments, the UCNP-substrates were blocked with 1 % BSA at room temperature.

### 4.9 NIR-controlled cell adhesion and spread in vitro

The disinfected UCNP-substrates were placed in 6-well plates, seeded with MSCs (10^5^ cells per well), and incubated for 6 h. The cells were then irradiated with near-infrared (NIR) light at various power levels (0, 0.5, 1, 2 W/cm^2^) for different durations (0, 20, 40, 60 min) in a darkroom. Following irradiation, the cell medium was washed with PBS and replaced with fresh medium. The cells were incubated for an additional 18 h. For the cell morphology experiment, the incubation period was extended to 24 h to allow better cell spreading. Subsequently, the MSCs were washed three times with PBS, fixed with 4 % paraformaldehyde, and stained with Actin-Tracker Green solution (1 % v/v) to visualize adhesion. After washing with PBS, cell adhesion and spreading were observed using an inverted fluorescence microscope.

### 4.10 Cell live/dead staining

MSCs (10^5^ cells) were seeded on the UCNP-substrates and subjected to NIR irradiation at different power levels (0, 0.5, 1, 2 W/cm^2^) for 40 min, then incubated for 48 h. Annexin V/PI kit testing solution were added to stain live and dead cells, respectively, and the cells were incubated for 20 min. After washing with PBS three times, the cells were imaged using an inverted fluorescence microscope to assess cell viability.

### 4.11 Immunofluorescence staining

The UCNP-substrate loaded with cells was extracted and fixed in paraformaldehyde. To facilitate antibody penetration into the cells, the sample was treated with Triton X-100. Blocking was performed using bovine serum albumin to minimize non-specific binding. The primary antibody was then added and incubated overnight at 4 °C. Following this, the sample was washed with phosphate-buffered saline (PBS) or alternative buffers to remove any unbound primary antibody. The fluorescently labeled secondary antibody was subsequently added, and incubation continued for 2 h at room temperature. After this incubation, the sample was washed again to eliminate any unbound secondary antibody. Finally, the fluorescent signal was observed and photographed using a fluorescence microscope. Images were acquired using a laser confocal microscope (Zeiss LSM 880, Germany).

### 4.12 RT-PCR

The UCNP-substrate in a 6-well plate was irradiated with near-infrared (NIR) light at different power levels (0, 0.5, 1, and 2 W/cm^2^) for 30 min. Following irradiation, the substrate was washed, disinfected, and MSCs were added for culture. After 48 h, the MSCs were induced to differentiate into osteoblasts and adipocytes using osteogenic and adipogenic differentiation media for 5 days. Subsequently, MSCs were washed with PBS three times and lysed with Trizol to extract total RNA for RT-PCR. For each sample, 1 μg of total RNA was reverse transcribed into cDNA using the First Strand cDNA Synthesis Kit. Approximately 2 μl of cDNA was then used for PCR amplification with a 2X PCR Master Mix. The forward and reverse primers used for amplification are listed in Table S1. PCR products were analyzed by 1 % agarose gel electrophoresis, with images captured using the ChemiScope 6000 (Clinx, Shanghai, China) and quantified with Image J.

### 4.13 Western blot

The UCNP-substrate in a 6-well plate was subjected to NIR irradiation at varying power levels (0, 0.5, 1, and 2 W/cm^2^) for 30 min. After irradiation, the substrate was washed, disinfected, and MSCs were added for culture. Following 48 h of culture, MSCs were induced to differentiate into osteoblasts and adipocytes using osteogenic and adipogenic media for 5 days. The MSCs were then washed with PBS three times and lysed to extract total protein for Western blot analysis. Protein concentration in each sample was measured using the BCA Protein Kit (Sangon, Shanghai, China). Approximately 30 μg of protein was mixed with 2x SDS-PAGE sample buffer and boiled for 5 min to denature the proteins. The samples were electrophoresed on a 12 % polyacrylamide gel and transferred to PVDF membranes (Immobilon, Sigma Aldrich, USA). Membranes were blocked with 5 % skim milk in TBST at room temperature, followed by incubation with primary antibodies in TBST overnight at 4 °C. Afterward, membranes were incubated with secondary antibodies for 1 h at room temperature, then subjected to enhanced chemiluminescence detection (ECL-Plus Kit, Solarbio, Beijing). Bands were visualized using the ChemiScope 6000 (Clinx, Shanghai, China) and quantified with Image J.

### 4.14 Alkaline phosphatase (ALP) and oil red O (OR) staining in vitro

The UCNP-substrate in a 6-well plate was irradiated with NIR light at varying power levels (0, 0.5, 1, and 2 W/cm^2^) for 30 min. After irradiation, the substrate was washed, disinfected, and MSCs were added for culture. After 48 h, MSCs were induced to differentiate into osteoblasts and adipocytes using osteogenic and adipogenic media for 7 days. The MSCs were then washed with PBS three times and fixed in 4 % paraformaldehyde. ALP and OR staining solutions (1 %) were applied for 1 h in the dark. Following staining, the UCNP-substrate in the 6-well plate was washed with PBS three times and photographed using an iPhone 12 and an optical microscope. Results were analyzed with Image J for statistical quantification.

### 4.15 Visualization of AAP-RGD release in vivo

Female CL6B57 mice, aged 6–8 weeks, were selected for the study. The mice received intraperitoneal injections of pentobarbital sodium to induce anesthesia. A 1-cm incision was made on the back to facilitate the implantation of the UCNP-substrate-FITC (5 × 5 mm), after which the incision was sutured. After completing the implantation procedure, the incision was sutured and maintained under sterile conditions. Following wound healing, the mice were re-anesthetized and randomly divided into four groups. The implantation sites were irradiated with near-infrared (NIR) light at varying power densities (0, 0.5, 1, and 2 W/cm^2^) for 40 min. The mice remained anesthetized throughout the irradiation period. Fluorescence and bright-field images of the dorsal region were acquired using a small animal imaging system. The in vivo release of AAP was evaluated based on the green fluorescence intensity of FITC.

### 4.16 Immunofluorescence staining in vivo

The preoperative procedure for the animal experiments was identical to that used in the visualization study. After natural wound healing, MSCs were precisely injected into the implantation site using a syringe (1 x 10^5^). The implantation sites of anesthetized mice were irradiated with 808 nm near-infrared (NIR) light at various power densities (0, 0.5, 1, and 2 W/cm^2^) for 40 min. For the subsequent 10 days, 100 μL of either osteogenic or adipogenic differentiation medium was injected daily into the UCNPS-substrate-implanted site to promote MSC differentiation. After 10 days, the UCNPS-substrate was retrieved and fixed with 4 % paraformaldehyde. The samples were permeabilized with Triton X-100 to enhance antibody penetration, followed by blocking with bovine serum albumin (BSA) to minimize nonspecific binding. Subsequently, primary antibodies targeting signaling pathway components or differentiation marker proteins were added and incubated overnight at 4 °C. After incubation, the samples were washed with PBS to remove unbound primary antibodies. The samples were then incubated with fluorescently labeled secondary antibodies at room temperature for 2 h. After incubation, the samples were washed again with PBS. Subsequently, cell nuclei were stained with DAPI. Fluorescence signals were then observed and imaged using a confocal microscope.

### 4.17 Immunohistochemistry (IHC)

The IHC staining procedure was largely like that of the immunofluorescence analysis. The primary distinction was in the choice of secondary antibodies. In this study, horseradish peroxidase (HRP)-conjugated secondary antibodies were employed. Following incubation with the primary and secondary antibodies and subsequent washing, color development was performed using a DAB substrate kit. Nuclei were then counterstained with hematoxylin. Images were acquired using an Olympus optical microscope, and the positive staining area was quantitatively analyzed using ImageJ software.

### 4.18 In vivo ALP and OR staining

After retrieval and fixation of the UCNP-substrate from the mice, ALP and OR staining was performed following the same procedure as used for in vitro assays.

### 4.19 Statistical analysis

All the experiments carried out in this report were repeated at least 3 times. All the statistical analyses were conducted using SPSS software (version 10; IBM Corp., Armonk, NY, USA). Different alphabet letters signify significant differences based on the statistical analyses (*p* < 0.05). Multiple group comparisons were performed using one-way analysis of variance (ANOVA), followed by post-hoc tests comparing each group mean to the 0 W/cm^2^ control group mean.

## CRediT authorship contribution statement

**Jinming Li:** Writing – review & editing, Supervision, Project administration, Investigation, Funding acquisition, Conceptualization. **Qingxin Zhao:** Writing – original draft, Software, Methodology, Formal analysis, Data curation. **Jiani Sun:** Data curation. **Hao Zeng:** Data curation. **Anli Yang:** Visualization, Funding acquisition.

## Ethics approval and consent to participate

All animal experiments were approved by the Institutional Animal Care and Use Committee, South China Normal University (Ethical number: SCNU-BIP-2024-014).

## Declaration of competing interest

The authors declare no conflict of interest.

## Data Availability

Data will be made available on request.
