# Supplementary material for: Near-infrared light-responsive upconversion substrate enables spatiotemporal control of mesenchymal stem cells adhesion and multilineage differentiation in vivo
Source: Mater Today Bio. 2025 Dec 26;36:102696. doi: 10.1016/j.mtbio.2025.102696 (PMC12813333; doi:10.1016/j.mtbio.2025.102696)
Supplement: Multimedia component 1 [file mmc1.docx]

**Supporting Information**


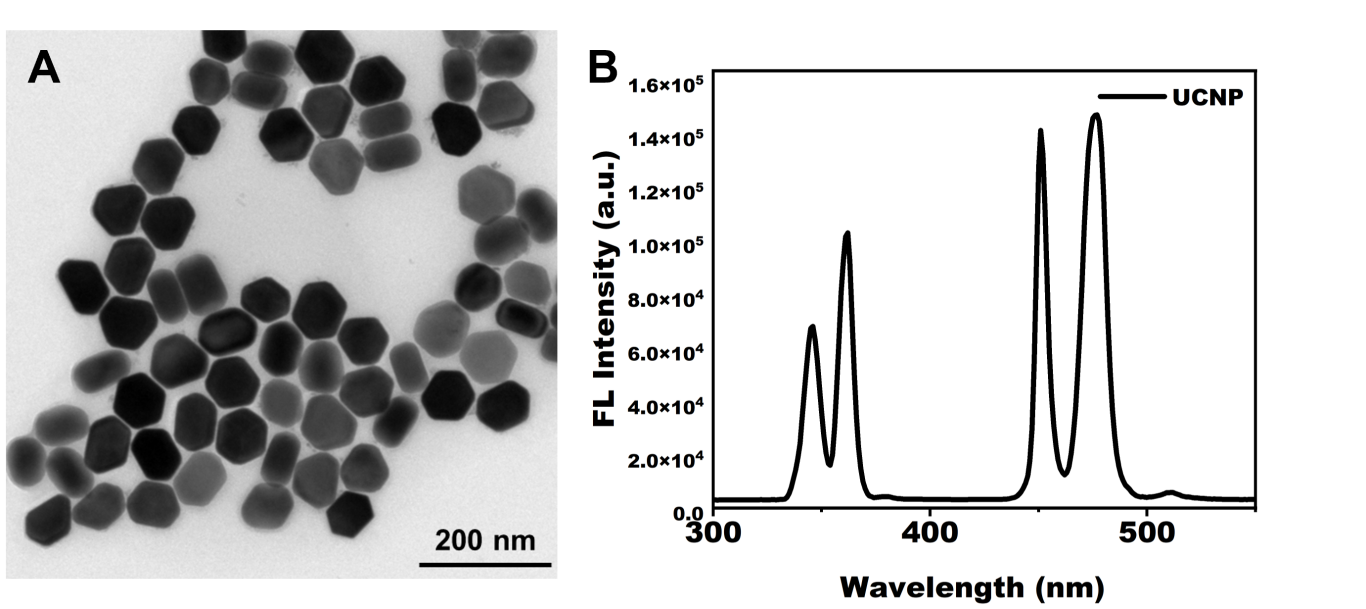


**Figure S1. Characterization of Core-Shell Tm@Nd UCNPs (NaYF₄:Yb/Tm@NaYF₄:Yb/Nd)**. (A) TEM image of the core-shell UCNPs, displaying a dumbbell-like structure with hexagonal cross-section and an average size of 150 nm ± 3.5 nm. (B) Fluorescence emission spectrum of the core-shell UCNPs under 808 nm NIR irradiation (1 W/cm²), showing characteristic UV emissions of Tm³⁺ ions (345 nm: ¹I₆ → ³F₄; 368 nm: ¹D₂ → ³H₆).


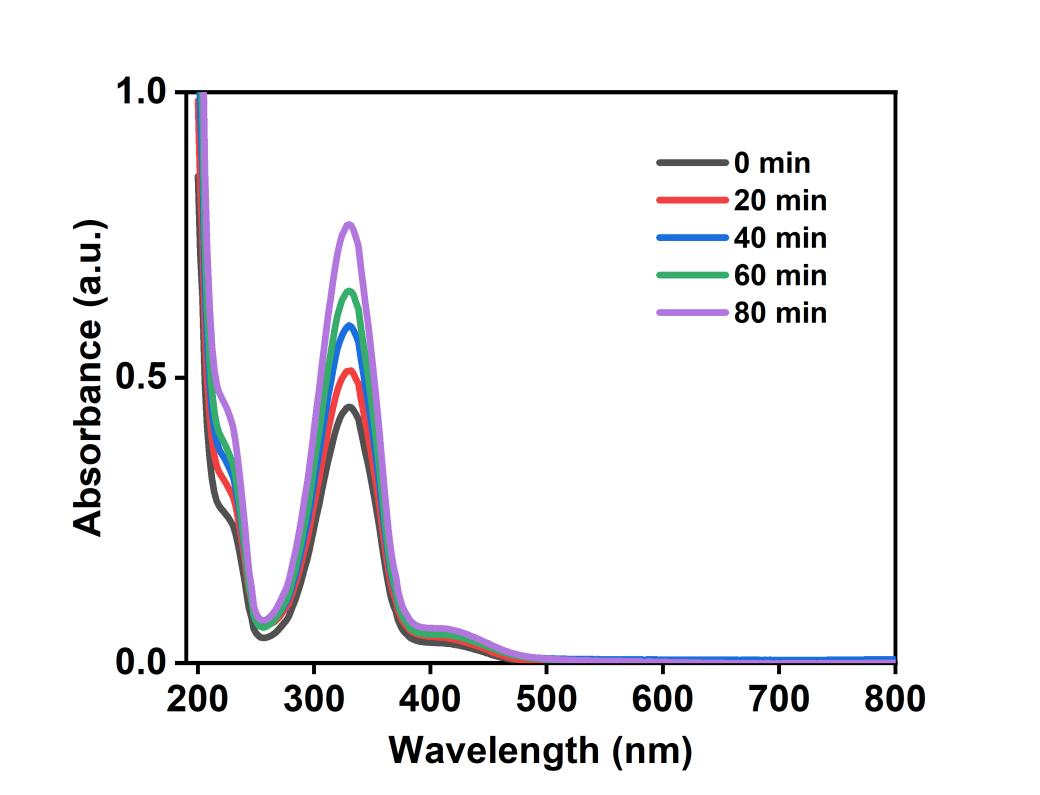


**Figure S2. Time-Dependent Release of AAP-RGD from Functional UCNPs**. Quantification of AAP-RGD release from UCNP@SiO₂-CD/AAP-RGD under continuous 808 nm NIR irradiation (1 W/cm²) over time (0-80 min), demonstrating a time-dependent increase in release.


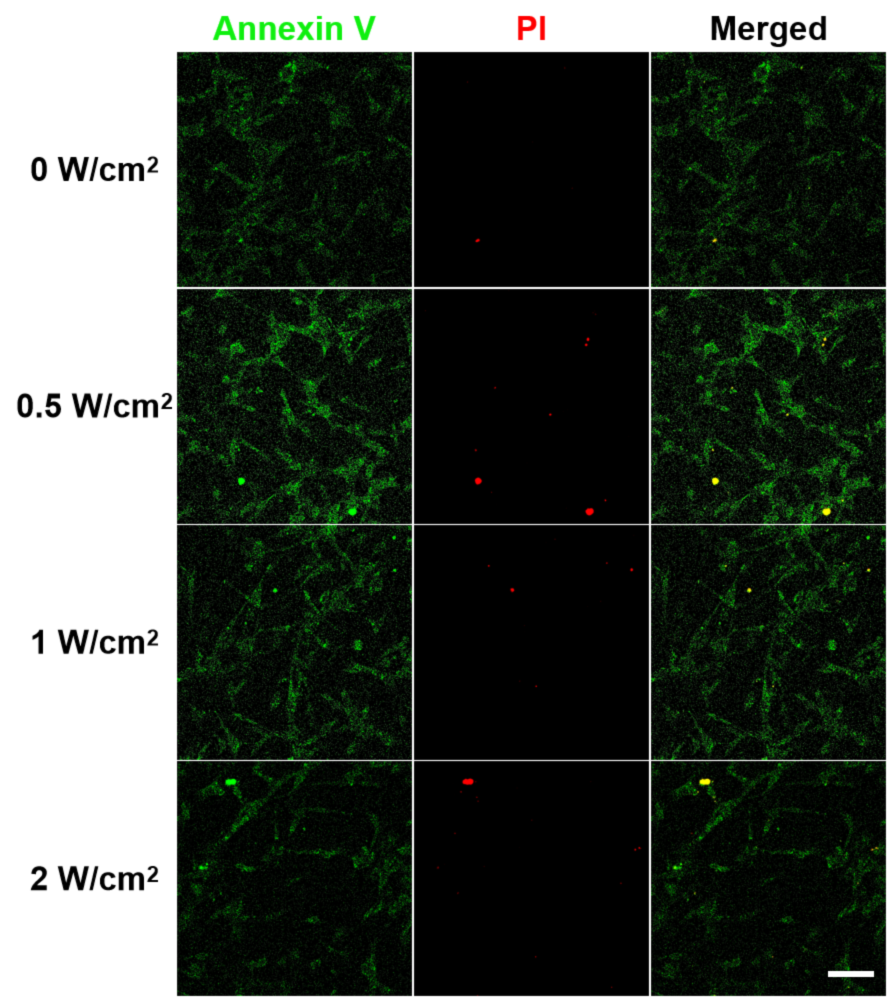


**Figure S3. Cytocompatibility Assessment of the UCNP-Substrate**. Representative live/dead (Annexin V-FITC/PI) staining images of MSCs cultured for 48 h on UCNP-substrates pre-exposed to different 808 nm NIR power densities (0-2 W/cm², 40 min). Scale bar: 20 µm. Results indicate no significant NIR-induced cytotoxicity under the tested conditions.


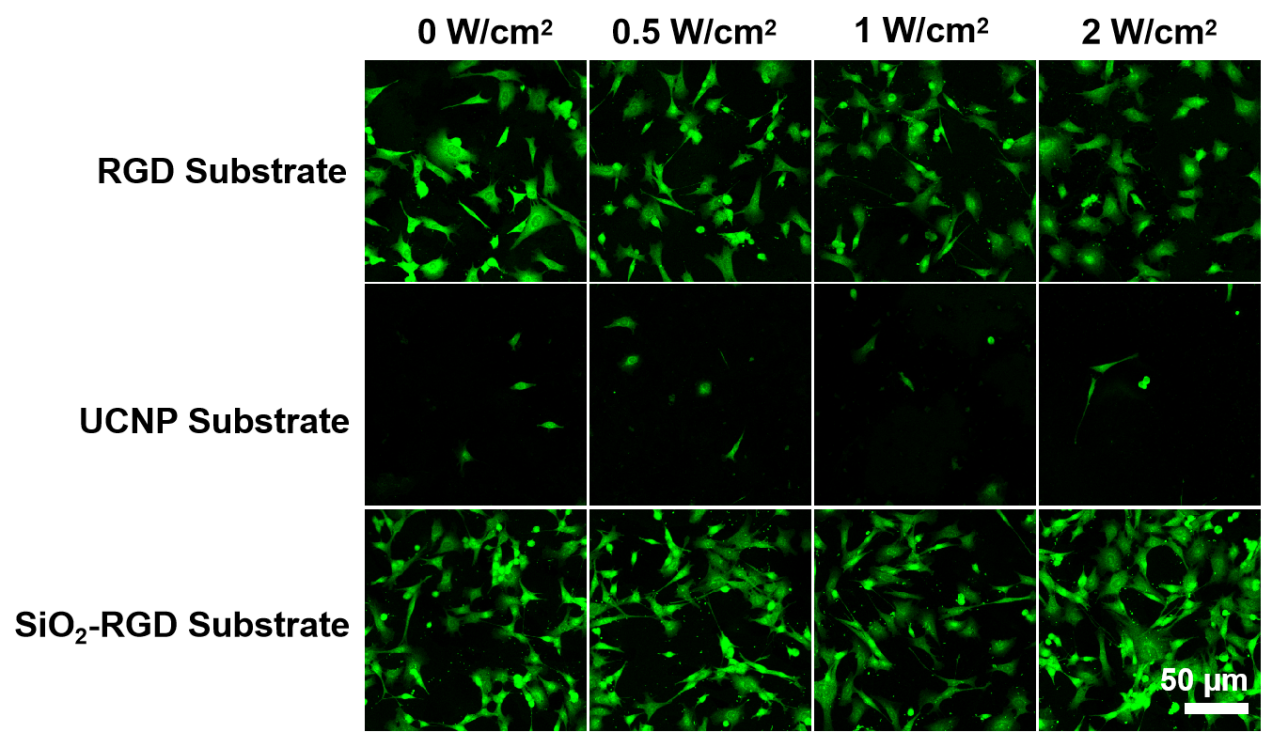


**Figure S4. Control Substrate of RGD Modification, UCNP Modification (no RGD on the surface) and SiO_2_-RGD Modification for Cell Adhesion Study.** RGD modification substrate showed a significant cell adhesion on substrate with the RGD peptide adsorption effect, and the UCNP substrate showed a very small number of cells adhered due to the absence of RGD modification on the surface of UCNPs. The SiO_2_-RGD substrate also showed a significant cell adhesion on substrate due to the absence of RGD modification on the surface of SiO_2_.


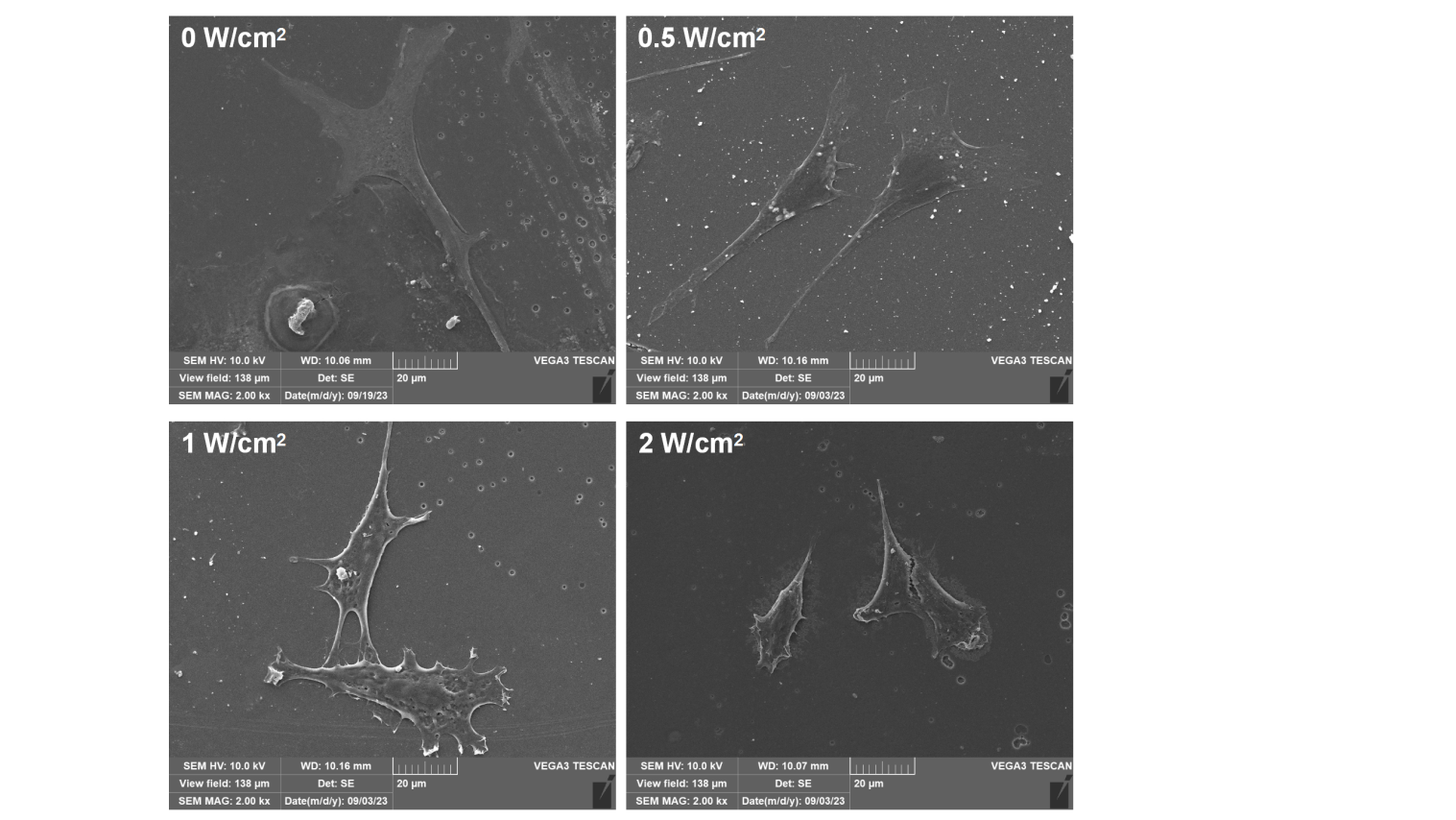


**Figure S5. SEM Analysis of MSC Adhesion and Spreading on NIR-Treated UCNP-Substrates**. Representative SEM images of MSCs cultured for 24 h on UCNP-substrates pre-treated with different 808 nm NIR power densities (0-2 W/cm², 40 min). Images illustrate the morphological transition from spread (0 W/cm²) to rounded (2 W/cm²) with increasing NIR power. Lower panel: Quantification of projected cell area.


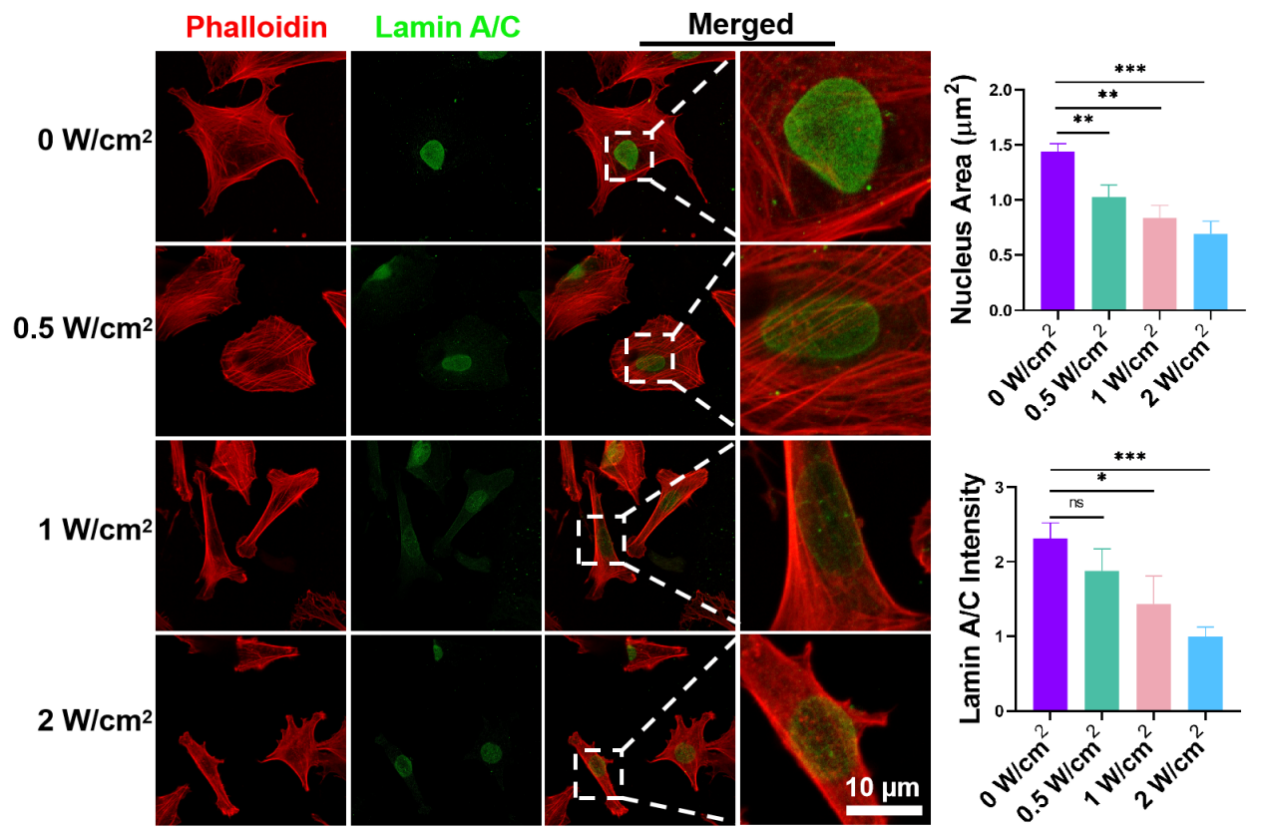


**Figure S6. NIR Regulation of Actin Cap Organization, Nuclear Morphology, and Lamin A/C Expression**. Immunofluorescence staining of F-actin (phalloidin, red), Lamin A/C (green), and nuclei (DAPI, blue) in MSCs cultured on UCNP-substrates pre-treated with different NIR power densities (0-2 W/cm², 40 min) for 24 h. Low-power NIR (0-0.5 W/cm²) promoted dense and aligned actin cap fibers, smooth nuclear morphology, and high Lamin A/C expression. High-power NIR (1-2 W/cm²) resulted in disordered/disrupted actin caps, wrinkled nuclei, and reduced Lamin A/C expression, indicating decreased nuclear stiffness. Scale bar: 10 µm. Lower panel: Quantification of nuclear circularity and Lamin A/C fluorescence intensity. Data represent mean ± s.e.m. (n = 3; *p<0.05, **p<0.01, ***p<0.001; N.S. = not significant).


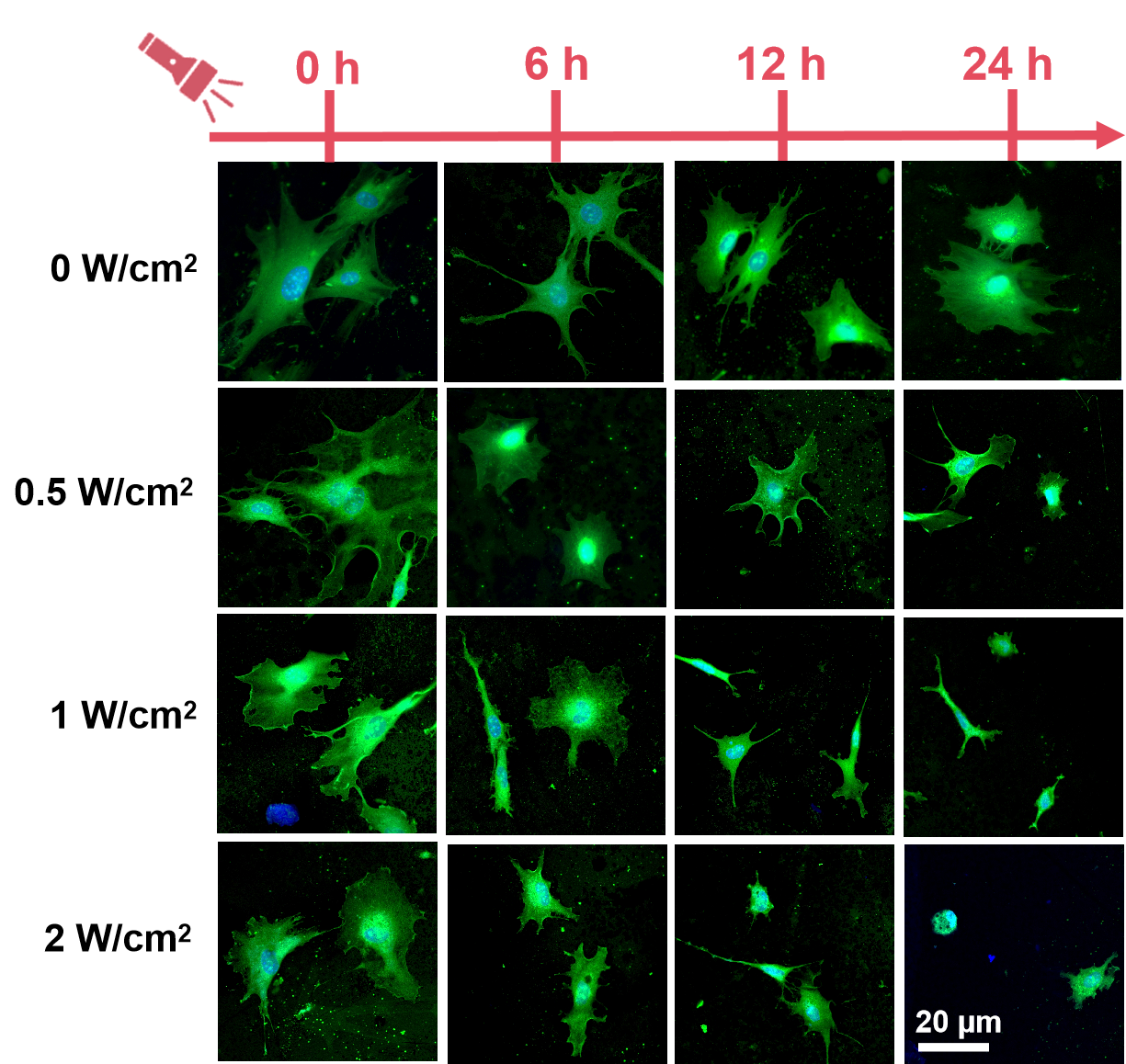


**Figure S7. Real-Time NIR Control of MSC Spreading Morphology**. Confocal microscopy images (F-actin, phalloidin-red; nuclei, DAPI-blue) of MSCs cultured on UCNP-substrates, subsequently exposed to localized 808 nm NIR irradiation at 0 W/cm² or 2 W/cm² for 40 min, and then further cultured for 24 h. Continuous NIR irradiation at 2 W/cm² induced a dynamic change from a spread, flat morphology to a rounded morphology, attributed to on-demand AAP-RGD detachment triggered by UCNP-upconverted UV light. Scale bar: 20 µm.


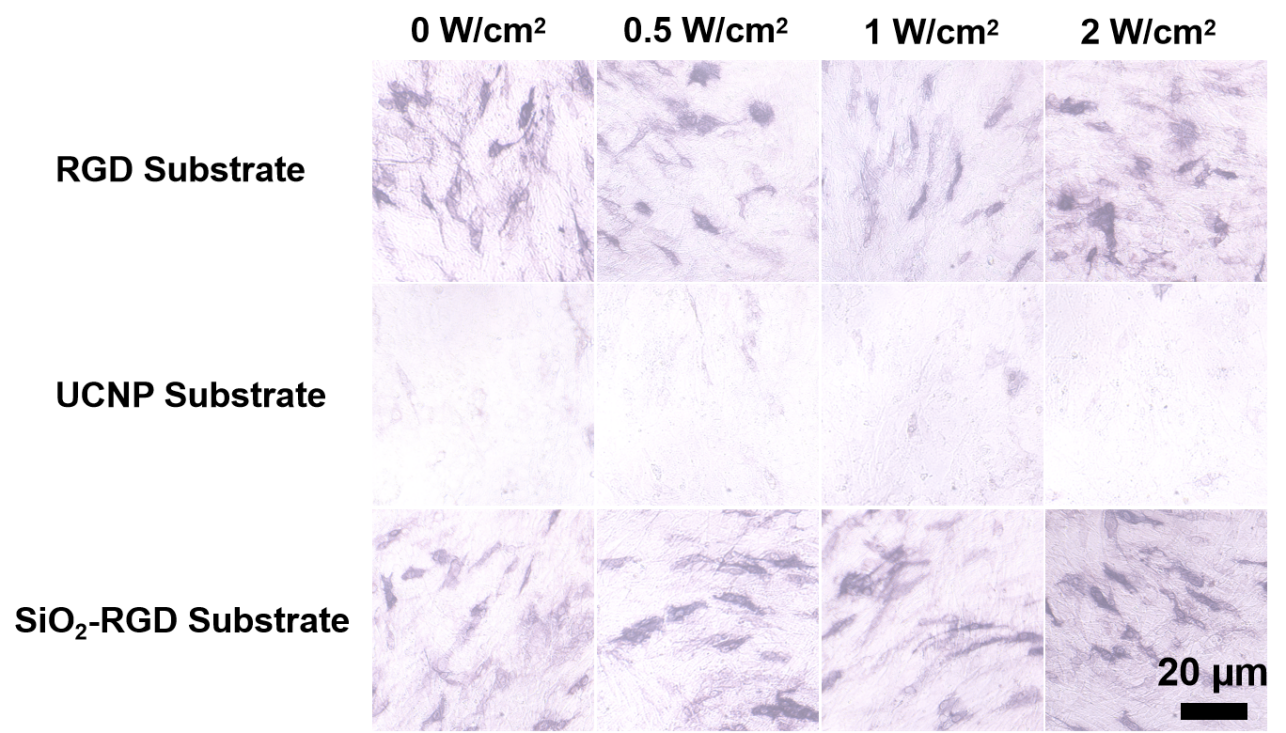


**Figure S8. Osteogenic Differentiation of MSCs on Various Control Substrates with ALP Staining.** The RGD-modified substrate exhibited significant ALP staining, attributed to the adsorption effect of the RGD peptide. In contrast, the UCNP substrate demonstrated minimal ALP staining due to the absence of RGD modification on its surface. Similarly, the SiO2-RGD substrate displayed substantial ALP staining as a result of the presence of RGD modifications. Although cells showed considerable ALP staining on both the RGD-modified and SiO2-RGD substrates, variations in NIR light power did not influence ALP staining on these substrates since there were no UCNPs present to modulate the amount of available RGD on their surfaces.


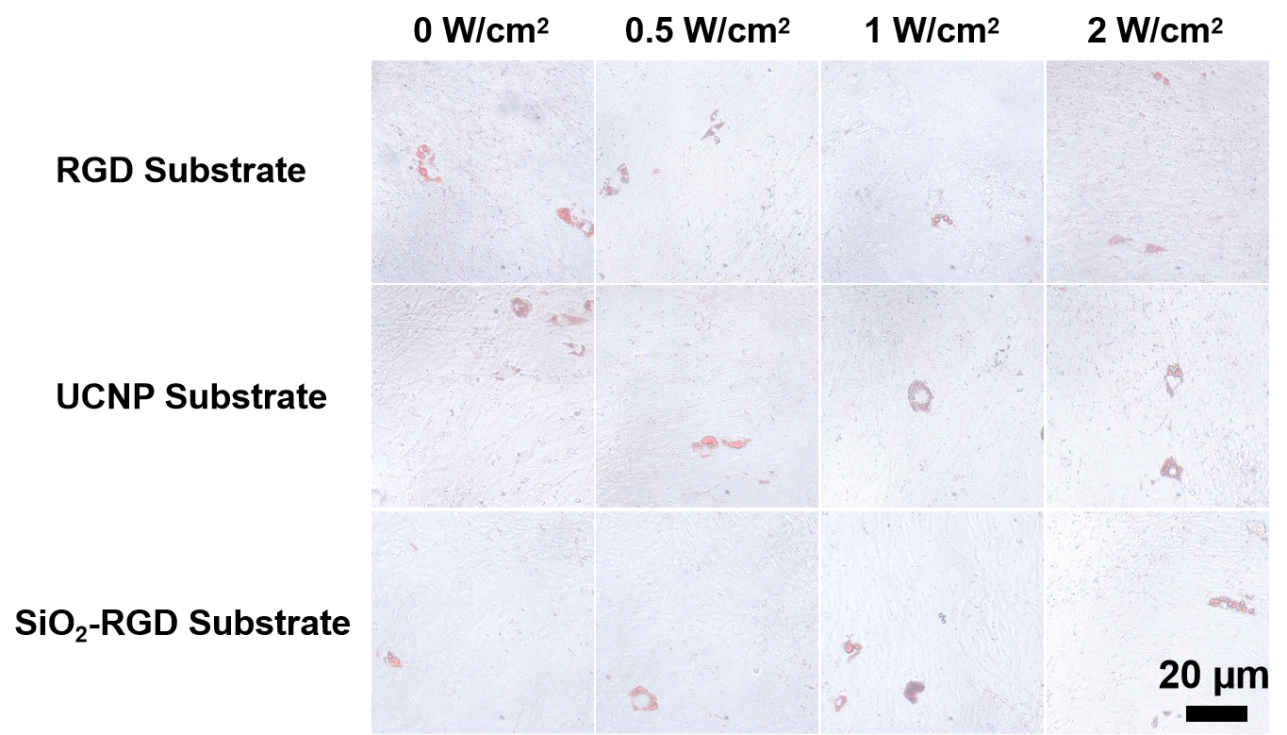


**Figure S9. Adipogenic Differentiation of MSCs on Various Control Substrates with Oil Red O Staining.** The results of Oil Red O staining were negligible across all control groups, as the adhesion force mediated by RGD prompted the cells to adopt a relatively flattened morphology on the substrates. Consequently, MSCs exhibited a greater propensity to differentiate into osteoblasts rather than adipocytes.


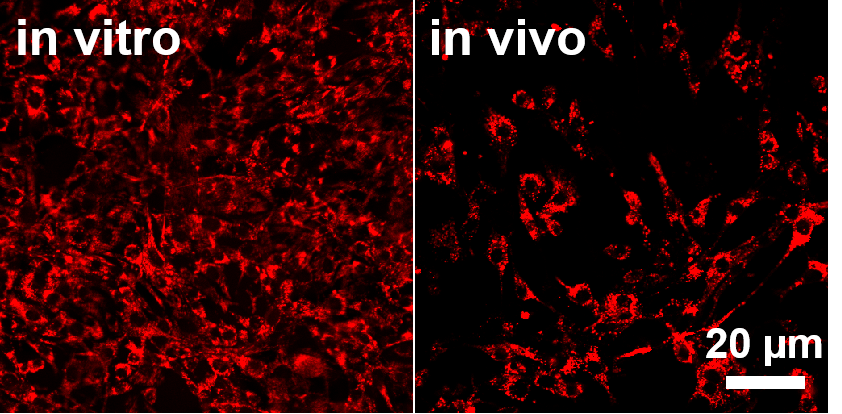


**Figure S10. Fluorescence Staining Images of MSCs in vivo and in vitro.** MSCs were labeled in vitro with the red fluorescent membrane dye PKH26. Successful incorporation of the dye was confirmed by fluorescence microscopy and then were detached, collected, and administered via subcutaneous injection at the substrate implantation site. Following a designated implantation period, the substrates were harvested, rinsed thoroughly (e.g., with PBS) to remove non-adherent cells, and subsequently analyzed by fluorescence microscopy (in vivo).

Table S1. The sequence and annealing temperature of primers used in RT-PCR.

| Gene | NCBI number | Forward (5’-3’) | Reverse (5’-3’) | Annealing temperature |
| --- | --- | --- | --- | --- |
| Runx2 | NM_001278483.1 | AACGATCTGAGA  TTTGTGGGC | CCTGCGTGGG  ATTTCTTGGTT | 57 ℃ |
| ALP | NM_001287172.2 | CCAACTCTTTTGTG  CCAGAGA | GGCTACATTGGTGT  TGAGCTTTT | 55 ℃ |
| OPN | NM_012881.2 | CTCCCGGTGAA  AGTGACTGA | TGCTTCTGAGAT  GGGTCAGG | 57 ℃ |
| C/EBPα | NM_007678.4 | CAAGAACAGCA  ACGAGTACCG | GTCACTGGTCAA  CTCCAGCAC | 56 ℃ |
| FABP4 | NM_001409513.1 | AAGGTGAAGAGCAT  CATAACCCT | TCACGCCTTTCATA  ACACATTCC | 55 ℃ |
| ADIPOQ | NM_009605.5 | ACGTCATCTTCG  GCATGACT | CTCTAAAGATTGTC  AGTGGATCTG | 56 ℃ |
